# A Biofabricated Human Acinus‐on‐a‐Chip Unveils Mechanotransductive Drivers of Ventilator‐Induced Lung Injury via Decoupling Volutrauma and Barotrauma

**DOI:** 10.1002/advs.77244

**Published:** 2026-08-17

**Authors:** Heng Lu, Liansheng Liu, Zengyong Hu, Shuxian Fang, Ruohan Ren, Sidi Liu, Meng Zhao, Zhiting Luo, Ziyue Zhang, Wenlong Shao, Qian Liu

**Affiliations:** ^1^ Department of Detection and Diagnosis Technology Research Guangzhou National Laboratory Guangzhou Guangdong People's Republic of China; ^2^ School of Biomedical Engineering Guangzhou Medical University Guangzhou People's Republic of China; ^3^ Micro‐nano Tech Center Bioland Laboratory Guangzhou Guangdong People's Republic of China; ^4^ The First Affiliated Hospital of Guangzhou Medical University Guangzhou Guangdong People's Republic of China

**Keywords:** acinus‐on‐a‐chip, biomimetics, disease models, mechanical injury decoupling, ventilator‐induced lung injury

## Abstract

Ventilator‐induced lung injury (VILI), an iatrogenic complication of mechanical ventilation, remains poorly understood due to the absence of physiologically relevant in vitro models, impeding targeted therapy development. Here, we engineer a bioinspired acinus‐on‐a‐chip model recapitulating human pulmonary acinar architecture and VILI pathological hallmarks, enabling mechanistic elucidation by decoupling distinct mechanical injury modes. Crucially, we uncover fundamentally divergent molecular pathways that volutrauma predominantly drives DNA damage response (DDR)‐mediated P53‐dependent apoptosis and nuclear factor kappa‐B (NF‐κB)‐driven inflammation, whereas barotrauma primarily triggers mitochondrial dysregulation, Wnt suppression, and metabolic collapse. Single‐cell profiling further reveals injury mode‐specific heterogeneity, identifying a rare TGF‐β‐enriched basal‐secretory transitional cluster in barotrauma that possibly drives fibrotic remodeling. Pharmacological P21 inhibition, agonist‐mediated Wnt pathway activation, or pathological mitochondrial fragmentation inhibition each significantly ameliorated VILI severity, with findings correlating strongly with clinical outcomes. Collectively, this study establishes a mechanobiological framework to optimize ventilation and reveals novel VILI therapeutics by integrating microengineering with stem cell biology.

## Graphical abstract

This bioinspired acinus‐on‐a‐chip recapitulates VILI pathology, revealing that volutrauma drives P53/NF‐κB pathways while barotrauma triggers mitochondrial‐Wnt dysregulation. A fibrotic transitional cell cluster was identified. Pharmacological interventions targeting these pathways significantly ameliorated injury, establishing a mechanobiological framework for ventilation optimization and therapy development.

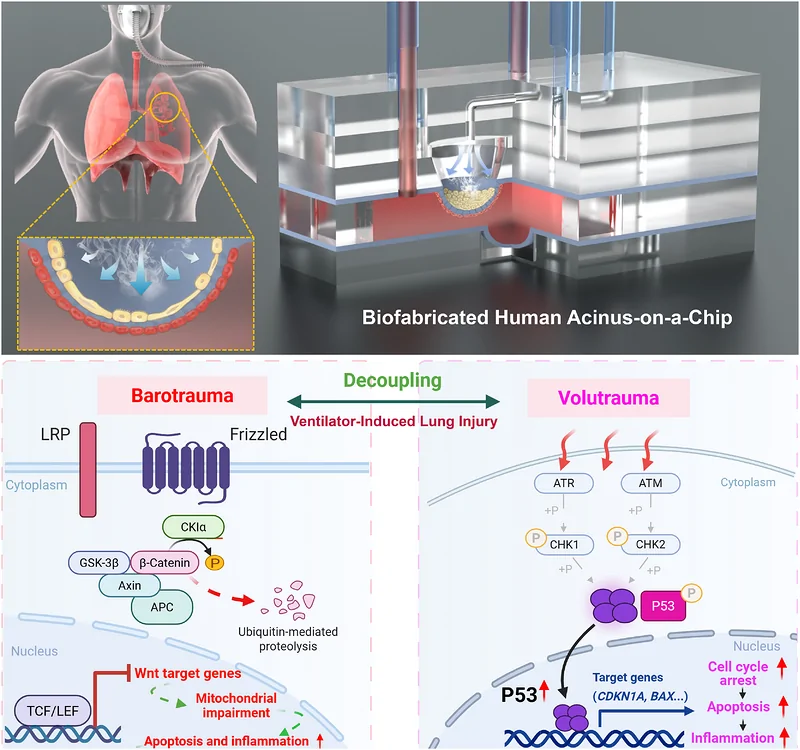

## 1 Introduction

The lungs, as vital organs, maintain systemic physiological homeostasis by regulating oxygen‐carbon dioxide exchange. Their direct exposure to airborne pathogens (bacteria, viruses), toxic gases, and particulate matter (PM2.5) makes them highly susceptible to inflammatory insults, frequently progressing to infections (e.g., pneumonia) or even acute respiratory distress syndrome (ARDS). ARDS stands as the leading cause of respiratory failure in critically ill patients, characterized by acute non‐cardiogenic pulmonary edema and hypoxemia secondary to air‐blood barrier disruption [1]. Mechanical ventilation (MV), a cornerstone therapeutic strategy for ARDS management [2, 3], is complicated by heterogeneous lung parenchymal pathology and mechanical stress at the interface between atelectatic and aerated lung regions. These factors increase tissue susceptibility to mechanical stretch during ventilation, exacerbating secondary injury and driving ventilator‐induced lung injury (VILI) [4, 5, 6, 7]. This process initiates a cascade of lung biotrauma, including damage‐associated molecular pattern (DAMP) release, alveolar‐capillary barrier breakdown (cadherin/occludin degradation), and mitochondrial reactive oxygen species (ROS) propagation, which subsequently instigates systemic inflammatory responses potentially progressing to multi‐organ failure, the primary mortality driver in ARDS [8]. While current primary strategies to reduce VILI risk through tailored positive end‐expiratory pressure and low tidal volume with reduced inspiratory pressures [9], the remaining complexity of VILI pathogenesis and the absence of reliable prevention strategies underscore the critical need to decipher injury mechanisms and translate these insights into clinically viable interventions for ARDS.

The initial pathogenesis of VILI is classically ascribed to four interconnected pathological mechanisms: biotrauma, barotrauma, volutrauma, and atelectrauma, which collectively drive lung injury through synergistic mechanical‐inflammatory crosstalk. Biotrauma, defined by the inflammatory cascade triggered by tissue injury, serves as a critical mediator of lung and multi‐organ failure in VILI. Ventilator‐induced alveolar hyperextension (volutrauma), excessive transpulmonary pressure (barotrauma), and cyclic alveolar opening‐closing (atelectrauma) act synergistically to initiate and amplify biotrauma, which not only perturbs alveolar epithelial and endothelial mechanotransduction but also disrupts critical homeostatic signaling pathways [10, 11]. These heterogeneous mechanics further destabilize the delicate balance between tissue repair and pathological remodeling. Evidence indicates that biotrauma in VILI is characterized by the induction of pro‐inflammatory mediator release and activation of canonical signaling pathways (e.g., NF‐κB, MAPK, PI3K/Akt), which synergistically promote reactive oxygen species (ROS) production and trigger inflammatory responses [11, 12]. Recently, researchers have gained deeper insights into the VILI‐associated biotrauma, revealing not only the release of key inflammatory mediators (e.g., TNF‐α, IL‐6, IL‐8) but also the dynamic infiltration and recruitment of monocytes/macrophages, leukocytes, and neutrophils. These processes orchestrate local inflammatory cascades and drive cell apoptosis, resulting in severe lung parenchymal injury, multi‐organ failure, and even mortality [12, 13, 14, 15, 16]. Despite extensive investigations into biotrauma mechanisms, the molecular underpinnings of volutrauma, barotrauma, and atelectrauma, as well as their intervening targeted therapeutic strategies, remain elusive, primarily due to the lack of reductionist experimental models capable of isolating and probing these distinct mechanobiological insults. Bridging this gap necessitates innovative platforms that precisely recapitulate the dynamic, context‐dependent mechanical environment of the injured lung tissue to dissect pathogenic pathways and accelerate translation.

Current investigations into VILI rely on three primary platforms: animal models, 2D cell stretch systems, and organ‐on‐a‐chip technologies, all of which have undergone significant advancements over the past decades. Animal models, while valued for their ability to recapitulate intact systemic physiological interactions, face practical limitations including prolonged experimental timelines, challenges in isolating individual mechanical variables, and ethical constraints that restrict their widespread use. 2D stretch systems, through enabling controlled exploration of cellular mechanoresponses, fail to reproduce the 3D architecture of the alveolar‐capillary unit, a critical limitation that constrains their utility in elucidating VILI pathogenesis. In contrast, organ‐on‐a‐chip systems represent a promising platform for in vitro modeling by integrating microphysiological 3D tissue interfaces with dynamic biomechanical forces, thereby enabling unprecedented fidelity in mimicking human organ pathophysiology. Of note, recent innovations in organ‐on‐a‐chip technology have further advanced VILI research: Sinem et al. engineered a bilayer lung‐on‐a‐chip featuring a nanofibrous membrane for applying physiological 2D stretch and model VILI‐relevant mechanical injury [17]; Vardhman et al. developed a microfluidic system utilizing basal air recirculation to induce 3D epithelial expansion across a flexible membrane, enabling biomechanical probing of VILI [18]; the Guenat group designed an alveolus‐on‐a‐chip maintaining air‐liquid interface (ALI) conditions to assess injury responses during 3D cyclic mechanical strain [19]; and Basia et al. introduced a ventilator‐on‐a‐chip platform integrating real‐time transepithelial/endothelial electrical resistance (TEER) monitoring to quantify barrier integrity dynamics [20]. Despite these strides in chip design, material optimization, and functional readouts, most studies have prioritized engineering innovation over mechanisms inquiry. A critical gap remains in elucidating the molecular pathways underlying atelectrauma, volutrauma, and barotrauma, particularly how specific mechanical insults (e.g., cyclic stretch, pressure gradients, alveolar reopening) translate into biological injury responses (e.g., inflammasome activation, barrier dysfunction, oxidative stress) remains poorly defined, limiting the translation of these models to actionable therapeutic strategies.

In this study, we engineered a high‐fidelity physiologically relevant human acinus‐on‐a‐chip model leveraging primary cell co‐cultures and programmable mechanical stress modulation to recapitulate volutrauma‐ and barotrauma‐induced air‐blood barrier disruption in VILI. Through systematically decoupling strain and pressure as independent mechanical variables and integrating transcriptomic profiling, we identified distinct pathological cascades driven by volutrauma vs. barotrauma in pulmonary acinar epithelium. Mechanistically, volutrauma preferentially activates DNA damage response (DDR) pathways, whereas barotrauma specifically perturbs Wnt/β‐catenin signaling and mitochondrial homeostasis. These divergent injury patterns converge to promote cell cycle arrest, apoptosis, and pro‐inflammatory activation, collectively exacerbating VILI progression. Single‐cell RNA sequencing further revealed transcriptional heterogeneity in injury responses, demonstrating that barotrauma uniquely induces a rare population of basal‐secretory transitional cell clusters with aberrant TGF‐β activation, suggesting barotrauma induces fibrotic remodeling. Notably, targeted therapeutic intervention including either DNA damage inhibition, Wnt/β‐catenin agonism, or pathological mitochondrial fission suppression significantly attenuated VILI‐associated pathology, underscoring the translational potential of targeting these mechanistically distinct pathways for clinical intervention.

## 2 Results

### 2.1 Biomimetic Engineering of a Human Lung Acinus‐on‐a‐Chip Reconstructs Pulmonary Acinar‐Capillary Barrier

Inspired by the lung's hierarchical, bifurcating architecture from air‐conducting zone (trachea, bronchi, terminal bronchioles) to the respiratory zone (respiratory bronchioles, alveolar ducts/sacs, alveoli), we developed a biomimetic, multilayered organ‐on‐a‐chip model to recapitulate the fundamental gas‐exchanging unit of pulmonary acinus (Figure 1a). The chip is constructed from multilayered polydimethylsiloxane (PDMS) via reverse‐molding and plasma bonding, with each layer possessing a unique structural configuration that collectively mimics the 3D architecture of the pulmonary acinus (Figure S1). The chip's side‐view illustrated a positive pressure‐driven air inlet for gas delivery to the acinar unit and a negative‐pressure‐driven 3D expansion mechanism for barrier deformation via the pneumatic unit (Figure 1b). COMSOL multiphysics simulations showed that, compared with the conventional chip (right), the trapezoidal geometry of our acinus‐on‐a‐chip (left) generates more complex unsteady flow patterns, including vortices and higher velocity, vorticity, and wall shear stresses (Figure 1c). Such flow features are also characteristic of pulmonary acinar environments [21, 22, 23], although quantitative differences exist due to dimensional and geometrical scaling. Laser profilometry quantified the 3D hemispherical deformation of the acinar barrier induced by pneumatic actuation (Figure 1d,e, Figure S2a, b, and Movie S1). Strain measurements showed that negative pressure at −200 mbar induced a maximal strain of ∼18%, whereas positive pressure at +200 mbar produced substantially less strain of ∼4% (Figure 1f), attributable to the significantly larger volume of the acinar region compared to the pneumatic chamber. This pressure‐dependent asymmetric deformation enables decoupling of distinct mechanical injury modalities in VILI. To validate the formation of a functional biological barrier within the acinar chip's respiratory microenvironment, we co‐cultured commercialized alveolar epithelial cells and pulmonary microvascular endothelial cells (PMECs) on opposing surfaces of a porous PDMS membrane to reconstruct the alveolar‐capillary barrier within the alveolar unit (Figure S2c). Immunofluorescence analysis confirmed proper polarization of the alveolar‐capillary interface, with alveolar epithelial cells and PMECs forming tight junctional complexes (E‐cadherin^+^ and VE‐cadherin^+^, respectively) in the acinar chips (Figure S2d and Movie S2).

**FIGURE 1:**
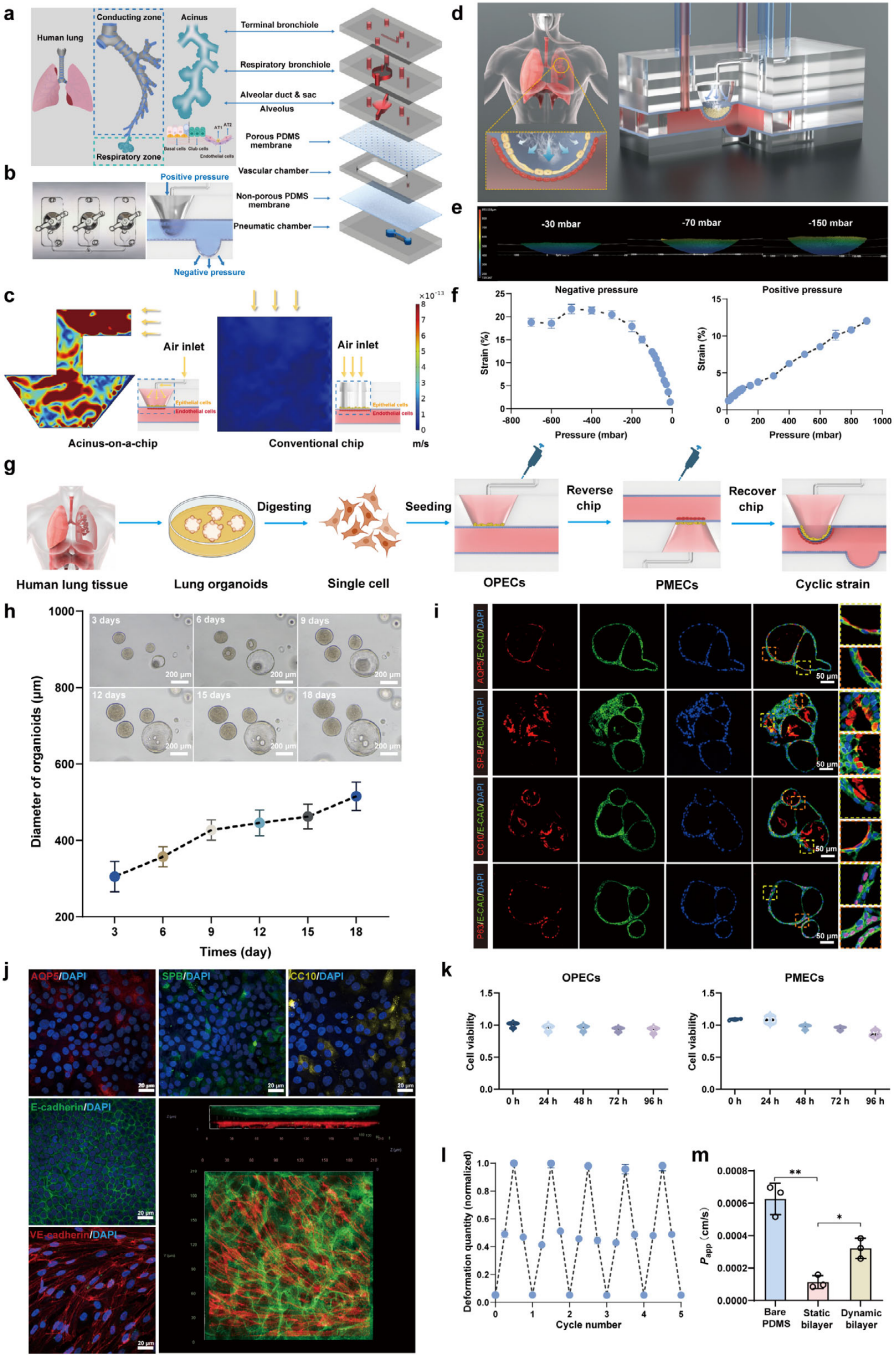
Design, fabrication, and functional validation of the human lung acinus‐on‐a‐chip. (a) Structural schematic of the acinus‐on‐a‐chip, designed to mimic the human lung's respiratory zone (respiratory bronchioles, alveolar ducts, sacs, and alveoli), comprising a heterogeneous population of airway and alveolar epithelial cells. (b) Photograph of the PDMS‐fabricated chip device and cross‐sectional schematic of the acinus‐on‐a‐chip, highlighting the distinct locations of positive pressure (PP) and negative pressure (NP). (c) COMSOL Multiphysics simulations of airflow velocity distribution in the acinus‐on‐a‐chip (left) and conventional chip (right) under equivalent positive‐pressure ventilation conditions. (d) Schematic of the acinus‐on‐a‐chip platform mimicking the air‐blood barrier and physiological respiratory motion. (e) 3D deformation images of the acinar unit at varying negative pressures (−30, −70, and −150 mbar), characterized by laser profilometry. (f) Strain of the acinar unit, calculated as (A‐A_0_)/A_0_ (A and A_0_ denote expanded and static surface areas), under variable negative and positive pressures. (g) Schematic workflow: adult stem cells, isolated from lung tissue of patients under approved ethical guidelines, were expanded to generate lung organoids. Organoid‐derived pulmonary epithelial cells (OPECs) and PMECs formed a polarized epithelial‐endothelial bilayer barrier in the chip, with pressure‐driven actuation mimicking pathophysiological 3D deformations. (h) Growth progression of pulmonary epithelial organoids from day 3 to day 18. (i) Confocal laser scanning microscopy (CLSM) images of paraffin‐embedded organoid sections depicting multicellular lineage composition. Alveolar type 1 (AT1) cells, alveolar type 2 (AT2) cells, club cells, and basal cells were labeled with aquaporin 5 (AQP5), surfactant protein B (SPB), club cell secretory protein 10 (CC10), and protein 63 (P63), respectively. Tight junctions (E‐cadherin, green) and cell nuclei (DAPI, blue) were immunolabeled. Scale bars: 50 µm. (j) CLSM images of the reconstructed epithelial‐endothelial barrier in the acinus‐on‐a‐chip, with OPECs and PMECs co‐cultured on opposite sides of the porous PDMS membrane. Scale bars: 20 µm. (k) Cell viabilities of OPECs and PMECs post‐barrier formation in the chip within 96 h. (l) Stability of the acinus‐on‐a‐chip under periodic strain (−70 mbar, 0.2 Hz). (m) The permeability of porous PDMS membranes in the chip under static and dynamic (cyclic strain: −70 mbar, 0.2 Hz) conditions before and after the construction of an epithelial‐endothelial bilayer barrier. The permeability was assessed by measuring transbarrier diffusion of Cascade Blue and 3 kDa Texas Red‐dextran. Data were presented as mean ± S.D., n = 3 biological replicates. Data were from one of three independent experiments. The data were analyzed by one‐way ANOVA with Tukey's multiple comparisons test (m). ^*^ *p* < 0.05, ^**^ *p* < 0.01.

The pulmonary acinus, as the distal gas‐exchange compartment extending from respiratory bronchioles to alveoli, exhibits specialized structural features supporting its complex epithelial ecosystem, which includes airway club cells, alveolar type 1 (AT1) cells, alveolar type 2 (AT2) cells, stem/progenitor cells, and transitional cell states [24, 25]. To recapitulate this cellular complexity in the chip, organoid‐derived pulmonary epithelial cells (OPECs) and pulmonary microvascular endothelial cells (PMECs) were co‐cultured on opposite sides of the porous PDMS membrane in the acinus‐on‐a‐chip to form a bilayer barrier recapitulating the acinar‐capillary interface (Figure 1g). Through systematic culture condition screening and optimization, we preserved characteristic vacuolar architecture and cellular diversity of distal lung organoids during expansion and passaging, enabling a more faithful recapitulation of the cellular diversity of airway and alveolar epithelial cells found in the native pulmonary acinar microenvironment (Figure 1h,i and Figure S2e). Immunofluorescence confirmed the multicellular lineage composition and intact barrier formation, with OPECs displaying complete E‐cadherin^+^ tight junctions and PMECs maintaining continuous VE‐cadherin adhesion (Figure 1j and Figure S2f). CCK‐8 assays confirmed sustained high viability (∼100% OPECs, ∼95% PMECs) and proliferative capacity over 96 h in the acinus‐on‐a‐chip (Figure 1k), demonstrating its capacity to support long‐term cellular homeostasis. Under periodic negative pressure (−70 mbar, 0.2 Hz), the epithelium‐endothelial barrier exhibited stable cyclic expansion and contraction with maintained structural integrity across multiple cycles (Figure 1l, Figure S2g, and Movie S3). Fluorescent tracer assays revealed the mechanically responsive barrier permeability regulation (0.0001 cm/s to 0.0003 cm/s, *p* < 0.05, under cyclic strain) (Figure 1m). Collectively, these results indicate that we present a bioinspired acinus‐on‐a‐chip that recapitulates the human pulmonary acinar‐capillary barrier, fluid dynamics, and cellular heterogeneity through advanced microfabrication and organoid technologies. By independently controlling air pressure and mechanical strain via selective pressure application, this system can decouple these typically interdependent variables, enabling unprecedented study of barotrauma and volutrauma during mechanical ventilation.

### 2.2 The Acinus‐on‐a‐Chip Recapitulates Pathophysiology of Ventilator‐Induced Lung Injury (VILI)

Leveraging the capabilities of controlled positive‐pressure ventilation and mechanical strain modulation in acinus‐on‐a‐chip, we established a VILI disease model. Cyclic strain of the porous membrane driven by negative pressure from the basal pneumatic chamber mimics 3D acinar deformation during spontaneous breathing, while positive pressure applied through the apical inlet replicates mechanical ventilation. Immunofluorescence analysis revealed stable expression of lineage markers (AQP5 for AT1 cells, SPB for AT2 cells, CC10 for club cells), but significant tight junction disruption with E‐cadherin (OPECs) and VE‐cadherin (PMECs) exhibiting fragmented pattern under cyclic assisted ventilation (AV, +20/‐70 mbar, 0.2 Hz) compared to static controls (Figure 2a). RT‐qPCR analysis revealed a significant upregulation of lineage‐specific markers (*AQP5*, *SFTPB*, *SCGB1A1*), suggesting a coordinated cellular stress response to mechanical injury that preserves lineage identity (Figure 2b). Under AV conditions, quantitative analysis showed modest declines in OPECs viability (∼100% to ∼75%), no significant alteration for PMECs viability (Figure 2c), and a 1.5‐fold increase in acinar‐capillary barrier permeability (0.00015 cm/s to 0.00038 cm/s, *p* < 0.01, Figure 2d). Consistent with clinical VILI pathogenesis, AV‐induced hallmark inflammatory responses displaying IL‐6 (105 to 161 pg/mL, ns), IL‐8 (1,132 to 7,558 pg/mL, *p* < 0.001), and TNF‐α (8.8 to 37 pg/mL, *p* < 0.001) levels surged, mirroring cytokine storms in ventilator‐associated ARDS patients (Figure 2e). Immunoblotting analysis revealed epithelial‐specific NF‐κB pathway activation during AV, with phosphorylated P65 (p‐P65) significantly elevated in OPECs (2.4 fold increase, *p* < 0.001) but no comparable change in PMECs, indicating preferential NF‐κB‐mediated epithelial injury (Figure 2f,g). These AV‐induced barrier disruption, pronounced cytokine secretion, and NF‐κB activation all validate the acinus‐on‐a‐chip's capacity to recapitulate VILI's pro‐inflammatory microenvironment and epithelial injury signatures.

**FIGURE 2:**
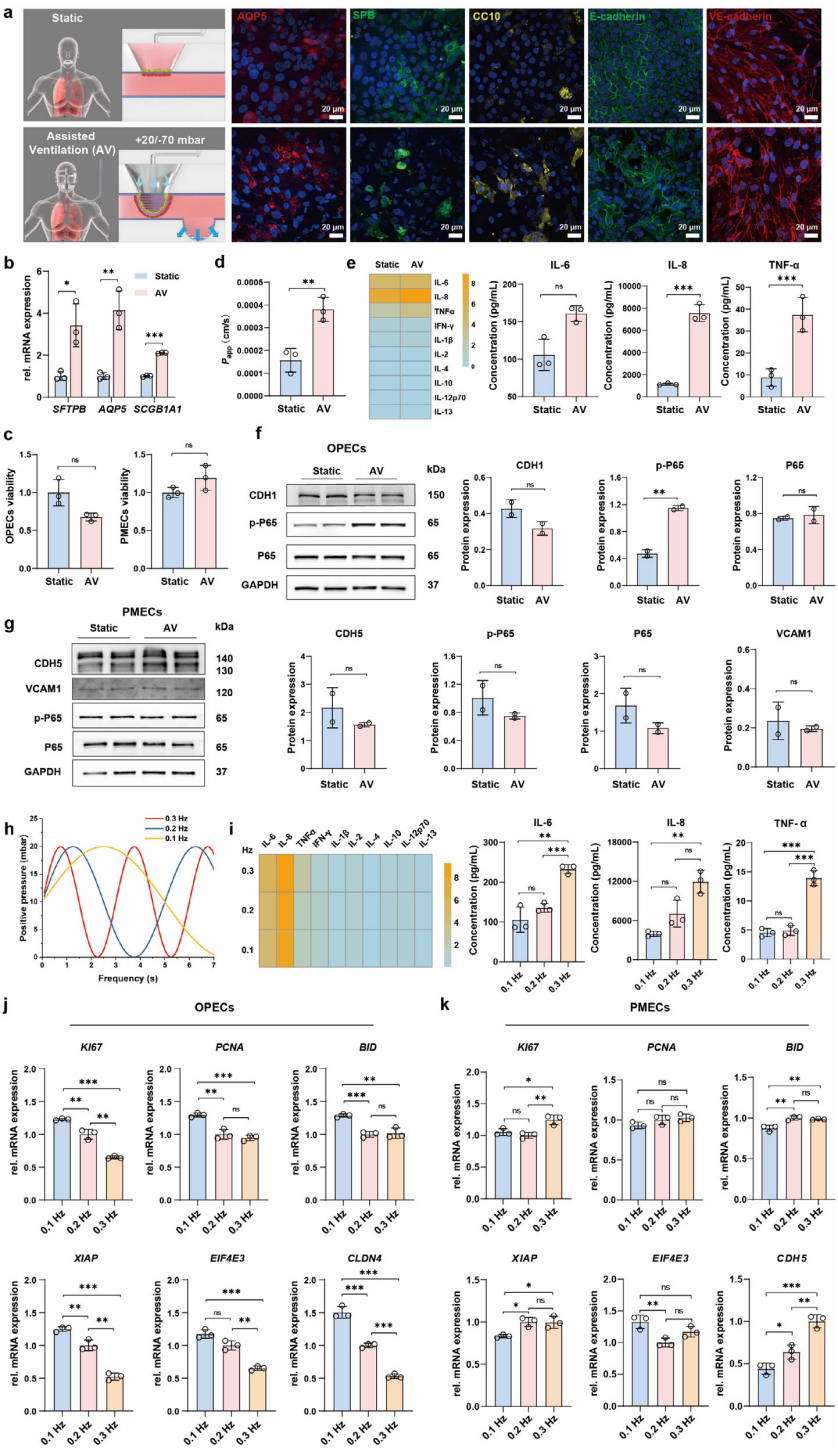
Pathological features of ventilator‐induced lung injury (VILI) recapitulated in the acinus‐on‐a‐chip. a. Left: Schematics of acinus‐on‐a‐chip under static conditions and assisted ventilation (AV: +20/‐70 mbar, 0.2 Hz). Right: Immunofluorescence characterization of cellular composition and barrier integrity, showing AT1 cells (AQP5, red), AT2 cells (SPB, green), club cells (CC10, yellow), tight junctions (E‐cadherin in OPECs, VE‐cadherin in PMECs), and nuclei (DAPI, blue). Scale bars: 50 µm. (b) RT‐qPCR analysis of OPECs markers (*AQP5*, *SFTPB*, *SCGB1A1*) under AV vs. static. (c) Cell viability of OPECs and PMECs under static and AV, quantified by CCK‐8 assay after 72 h culture. (d) Barrier permeability under static and AV, assessed by measuring trans‐barrier diffusion of Cascade Blue and 3 kDa Texas Red‐dextran. (e) Heatmap of cytokine profiles (IL‐6, IL‐8, TNF‐α, IFN‐γ, IL‐1β, IL‐2, IL‐4, IL‐10, IL‐12p70, IL‐13) in the acinus‐on‐a‐chip under static and AV conditions at 24 h, quantified by multiplex immunoassay. (f) Immunoblotting analysis of junctional proteins (CDH1) and NF‐κB signaling of total P65 and phosphorylated P65 (p‐P65) in OPECs under static and AV at 24 h. (g) Immunoblotting analysis of junctional proteins (CDH5), adhesion marker (VCAM1), total P65, and p‐P65 in PMECs under static and AV at 24 h. GAPDH served as a loading control. (h) Simulation of ventilator‐delivered different positive pressure frequencies (0.1, 0.2, 0.3 Hz) within the acinus‐on‐a‐chip model. (i) Heatmap of cytokine profiles in the acinus‐on‐a‐chip under mechanical ventilation across variable positive‐pressure frequencies (0.1, 0.2, 0.3 Hz, +20 mbar) with constant negative‐pressure baseline (0.2 Hz, ‐70 mbar). (j) RT‐qPCR of epithelial homeostasis gene expression (*KI67*, *PCNA*, *BID*, *XIAP*, *EIF4E3*, *CLDN4*) in OPECs at different positive‐pressure frequencies. (k) RT‐qPCR of endothelial homeostasis gene expression (*KI67*, *PCNA*, *BID*, *XIAP*, *EIF4E3*, *CDH5*) in PMECs at different positive‐pressure frequencies. Data were presented as mean ± S.D., n = 3 biological replicates. Data were from one of three independent experiments. The data were analyzed by two‐tailed Student's t‐test (c, d, e, f, g), one‐way ANOVA with Tukey's multiple comparisons test (i, j, k), and two‐way ANOVA with Tukey's multiple comparisons test (b). ^*^ *p* < 0.05, ^**^ *p* < 0.01, ^***^ *p* < 0.001, ns, not significant.

To investigate how ventilation frequency modulates VILI pathogenesis, we applied different positive‐pressure ventilations (0.1, 0.2, 0.3 Hz, +20 mbar) through the apical inlet while maintaining physiological spontaneous breathing (0.2 Hz, −70 mbar) via basal negative‐pressure actuation (Figure 2h). Figure 2i demonstrated that inflammatory markers (IL‐6, IL‐8, TNF‐α) remained at baseline levels under ventilation frequencies ≤ 0.2 Hz but exhibited significant upregulation beyond physiological respiratory rates, uncovering a critical frequency threshold for ventilator‐induced inflammatory activation. Quantitative mRNA levels revealed progressive downregulation of epithelial homeostasis genes at supraphysiological ventilation frequencies (0.3 Hz), including proliferation regulators (*KI67*), DNA replication factors (*PCNA*), apoptosis mediators (*BID*, *XIAP*), cell cycle controllers (*EIF4E3*), and barrier integrity proteins (*CLDN4*) (Figure 2j). This frequency‐dependent suppression of cellular repair mechanisms, combined with elevated inflammatory responses, established 0.2 Hz as the optimal ventilation frequency to minimize iatrogenic injury while preserving gas exchange efficiency. Elevated frequencies of positive‐pressure ventilation induced modest upregulation of PMECs proliferation (*KI67*, *MCM5/6*), while DNA replication factors (*PCNA*) remained unaffected (Figure 2k and Figure S3a). Concurrently, cell cycle regulators underwent modest transcriptional changes, with *EIF4E3* slightly downregulated and *CDKN1A* (a cell cycle inhibitor) upregulated; meanwhile, pro‐apoptotic genes (*BID*, *BAK*, *XIAP*, *ATG9B*, *FAS*) and the endothelial adhesion molecule *CDH5* showed consistently slightly elevated transcription (Figure 2k and Figure S3a). These results indicate that mismatched patient‐ventilator frequencies damage the epithelium more severely than the endothelium. Collectively, these results indicate that the acinus‑on‑a‑chip can recapitulate key features of VILI pathophysiology under clinically relevant ventilation, such as mechanical strain‑induced barrier dysfunction, inflammatory activation, and frequency‑dependent disruption of acinar‑capillary homeostasis.

### 2.3 Acinus‐on‐a‐Chip Decouples Distinct Mechanical Injury Modalities of Volutrauma and Barotrauma in VILI

In clinical practice, volutrauma (overdistension‐mediated lung injury) and barotrauma (high inflation pressure‐mediated lung injury) often occur simultaneously [26, 27]. To dissect the distinct contributions of volutrauma and barotrauma in VILI pathogenesis, we leveraged the acinus‐on‐a‐chip platform to independently modulate mechanical strain and inflation pressure, thereby isolating these two mechanical injury modalities. Volutrauma was modeled by applying negative pressures (V_‐70_, V_‐100_, V_‐200_) to the pneumatic unit, which drove the epithelial‐endothelial barrier into a 3D expansion corresponding to either physiological or pathological strain levels. Because the culture medium above the barrier was open to atmospheric pressure, the transpulmonary pressure remained essentially unchanged, thereby isolating strain‐induced injury as a proxy for volutrauma (Figure 3a). Barotrauma was modeled by delivering clinically relevant positive pressures (B_+20_, B_+30_, B_+40_) through the upper air inlet, imposing transpulmonary pressure‐like stress on the barrier. Under these conditions, the barrier experienced only ∼1.5% strain, well below the threshold for volutrauma, so the resulting injury could be attributed predominantly to stress‐induced barotrauma (Figure 3b). This experimental strategy thus enabled us to dissociate strain‑dependent volutrauma from stress‑dependent barotrauma and compare their individual contributions in a controlled in vitro system. Immunofluorescence analysis demonstrated that both volutrauma and barotrauma induced pressure‐dependent disruption of tight junction proteins (E‐cadherin/VE‐cadherin) at the acinar‐capillary interface. Volutrauma caused a marked reduction in viability of both OPECs and PMECs, whereas barotrauma predominantly impaired OPECs viability with minimal impact on PMECs (Figure 3c). Immunoblotting analysis demonstrated that only V_‐70_ significantly upregulated CC10 and AQP5 expression under varying pressures, suggesting that physiologically relevant respiratory movements stimulate club cell and AT1 cell differentiation (Figure 3d–g).

**FIGURE 3:**
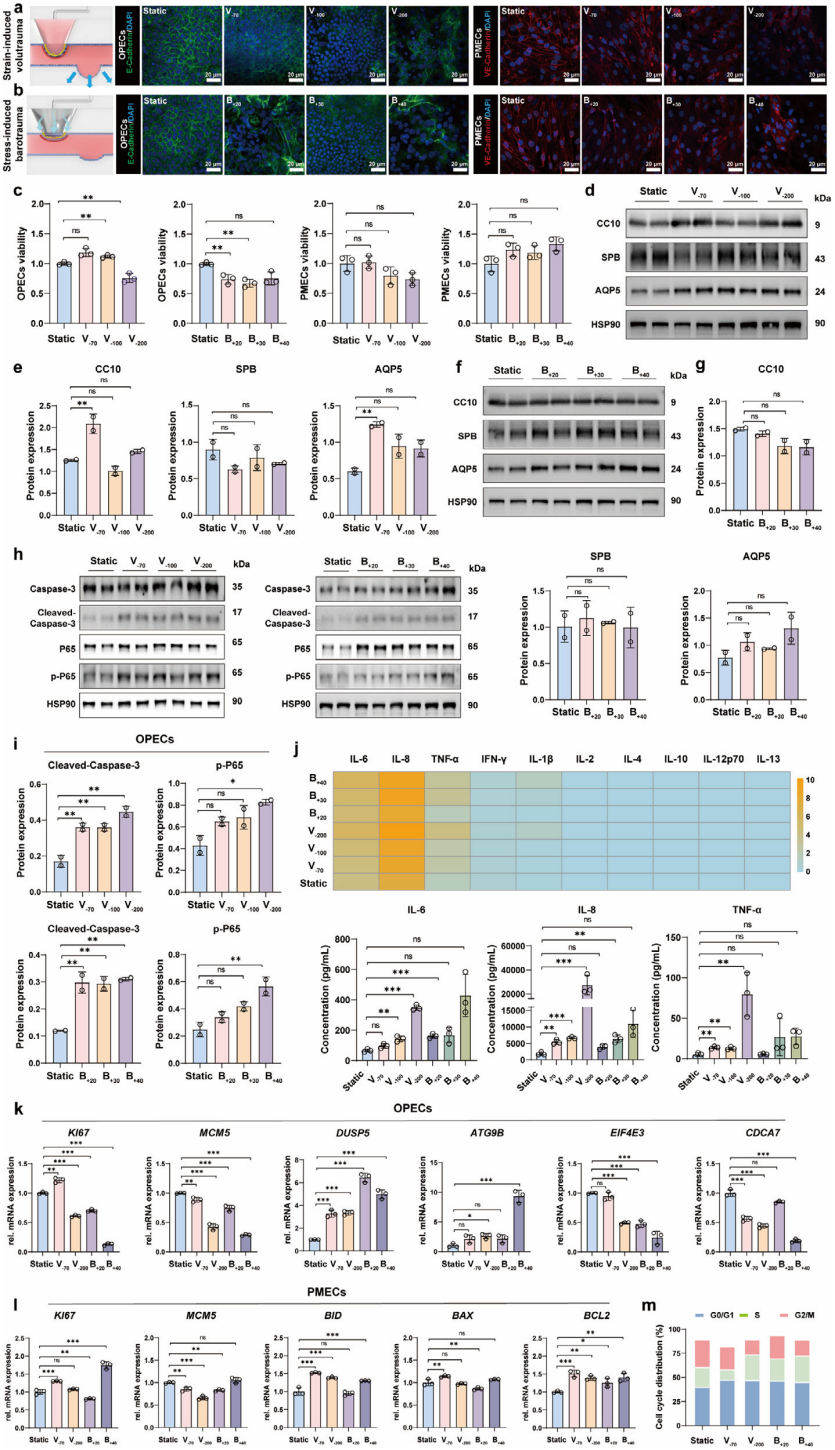
Mechanistic investigation of volutrauma‐ and barotrauma‐induced VILI using the acinus‐on‐a‐chip model. (a) Volutrauma simulation: (Left) schematic depicting acinar overdistension induced by negative pressures. (Right) Immunofluorescence of OPECs (E‐cadherin, red) and PMECs (VE‐cadherin, green) showing junctional integrity disruption under volutrauma (negative pressures: V‐_70_, V_‐100_, V_‐200_). (b) Barotrauma simulation: (Left) schematic of positive‐pressure ventilation with a low strain. (Right) Immunofluorescence of OPECs/PMECs demonstrating barrier disruption under barotrauma (positive pressures: B_+20_, B_+30_, B_+40_). Nuclei: DAPI (blue). Scale bars: 20 µm (all panels). (c) Pressure‐dependent viability changes (CCK‐8 assay) of OPECs and PMECs in the chips under volutrauma (0 to ‐200 mbar) and barotrauma (0 to +40 mbar). (d–g) Immunoblotting and quantified protein expression of OPECs markers (AQP5, SPB, CC10) in chips subjected to varied volutrauma (d, e) and barotrauma (f, g). (h) Activation of apoptotic (caspase‐3 cleavage) and inflammatory (P65 phosphorylation) signaling in OPECs during volutrauma (0 to ‐200 mbar) and barotrauma (0 to +40 mbar), and the corresponding quantified analysis (i). HSP90 was used as a loading control. (j) Multiplex immunoassay quantification of inflammatory cytokines (IL‐6, IL‐8, TNF‐α, IFN‐γ, IL‐1β, IL‐2, IL‐4, IL‐10, IL‐12p70, IL‐13) in volutrauma‐ and barotrauma‐induced acinus‐on‐a‐chip models. (k) RT‐qPCR analysis of proliferation markers (*KI67*, *MCM5*), apoptosis genes (*DUSP5*, *ATG9B*), and cell cycle regulators (*EIF4E3*, *CDCA7*) in OPECs at V_‐70_, V‐_200_, B_+20_, and B_+40_. (l) RT‐qPCR analysis of *KI67*, *MCM5*, *BID*, *BAX, BCL2* in PMECs at V_‐70_, V‐_200_, B_+20_, and B_+40_. (m) Flow cytometry histograms (PI staining) showing quantification of cell cycle phase distribution (G0/G1, S, G2/M) in OPECs under volutrauma and barotrauma. Data were presented as mean ± S.D., n = 3 biological replicates. Data were from one of three independent experiments. The data were analyzed by one‐way ANOVA with Tukey's multiple comparisons test (c, e, g, i, j, k, l). ^*^ *p* < 0.05, ^**^ *p* < 0.01, ^***^ *p* < 0.001, ns, not significant.

Notably, both injury modes triggered pressure‐dependent apoptotic activation of OPECs and PMECs (cleaved caspase‐3: increased to 2.6‐fold at V_‐200_, *p* < 0.01; increased to 2.4‐fold at B_+40_; *p* < 0.01) and inflammatory signaling (p‐P65: increased to 2.0‐fold at V_‐200_, *p* < 0.05; increased to 2.5‐fold at B_+40_, *p* < 0.01), with dose‐dependent injury severity (Figure 3h,i and Figure S3b, c). Cytokine profiling revealed pressure‐dependent increases in pro‐inflammatory mediators (IL‐6, IL‐8, TNF‐α), with the most pronounced inflammatory responses at V_‐200_ (IL‐6: 5.2‐fold, IL‐8: 14.5‐fold, TNF‐α: 15.9‐fold; all *p* < 0.01), suggesting the alveolar overdistension in volutrauma may drive more severe inflammation than barotrauma (Figure 3j). Both volutrauma and barotrauma impaired OPECs proliferation, as evidenced by downregulated proliferative genes (*KI67*, *MCM5*) (Figure 3k). Apoptosis‐related genes (*DUSP5*, *ATG9B*) were upregulated, and cell cycle regulators (*EIF4E3*, *CDCA7*) were downregulated in a dose‐dependent manner (Figure 3k). In contrast, PMEC cells in the acinus‐on‐a‐chip showed minor changes in proliferation and apoptosis under cyclic positive and negative pressure stimulations (Figure 3l). It implies mechanical ventilation inflicts more severe epithelial damage than endothelial injury. Flow cytometry further demonstrated cell cycle disruption exhibiting that both volutrauma and barotrauma increased G0/G1 (V_‐200_: 40% to 47.1%; B_+40_: 40% to 45.3%) and S phases (V_‐200_: 20.7% to 26.8%; B_+40_: 20.7% to 27.4%) populations while depleting G2/M phase (V_‐200_: 28.7% to 15.6%; B_+40_: 28.7% to 16.5%), a hallmark of VILI pathogenesis (Figure 3m and Figure S3d). Collectively, the acinus‐on‐a‐chip platform appears to effectively decouple key pathophysiological features of volutrauma and barotrauma, revealing that mechanical ventilation‐induced acinar overdistension (volutrauma) and high pressure (barotrauma) preferentially trigger acinar epithelial apoptosis and cell cycle dysregulation, with volutrauma driving more severe acute inflammation, an observation that warrants further mechanistic investigation of underlying pathways.

### 2.4 Transcriptomic Profiling Implies Distinct Molecular Injury Pathways Between Volutrauma and Barotrauma

To delineate the molecular mechanisms of ventilator‐induced barrier dysfunction, we performed RNA sequencing (RNA‐seq) on OPECs and PMECs in the acinus‐on‐a‐chip following controlled volutrauma (V_‐70_ and V_‐200_) and barotrauma (B_+20_ and B_+40_) exposure. Heatmap analysis revealed distinct transcriptional profiles in OPECs and PMECs exposed to volutrauma (V_‐70_ and V_‐200_) vs. static conditions (Figure 4a and Figure S4a). Gene Ontology (GO) enrichment of differentially expressed genes (DEGs) in OPECs showed that downregulated genes at V_‐200_ were enriched in genome maintenance pathways (DNA replication, double‐strand break repair, and homologous recombination), while upregulated genes were linked to acute‐phase responses (inflammatory signaling, wound healing, and innate immunity) (Figure 4b,c). Transcriptomic analysis revealed different gene expression patterns in PMECs, with V_‐200_ upregulating cell‐junction and differentiation genes while downregulating DNA replication regulators (Figure S4b, c). Gene set enrichment analysis (GSEA) demonstrated significant enrichment of cytokine‐cytokine receptor interaction and immune response pathways in V_‐200_‐treated PMECs (Figure S4d). We further identified 260 consistently upregulated genes and 198 downregulated genes across both V_‐70_ and V_‐200_ compared to static controls in OPECs (Figure 4d). The upregulated genes were primarily involved in lung development, apoptosis, and DNA damage processes, while the downregulated genes were associated with DNA recombination, cell cycle, and double‐strand break repair (Figure 4e). GSEA revealed that volutrauma‐treated OPECs upregulated inflammatory/apoptosis genes but downregulated cell cycle pathways (E2F targets, G2M checkpoint) (Figure 4f). Taken together, these findings suggest that volutrauma induces acinar epithelial injury through converging mechanisms of proliferation arrest, inflammatory activation, and DNA damage accumulation, while exerting differential pathophysiological effects on PMECs.

**FIGURE 4:**
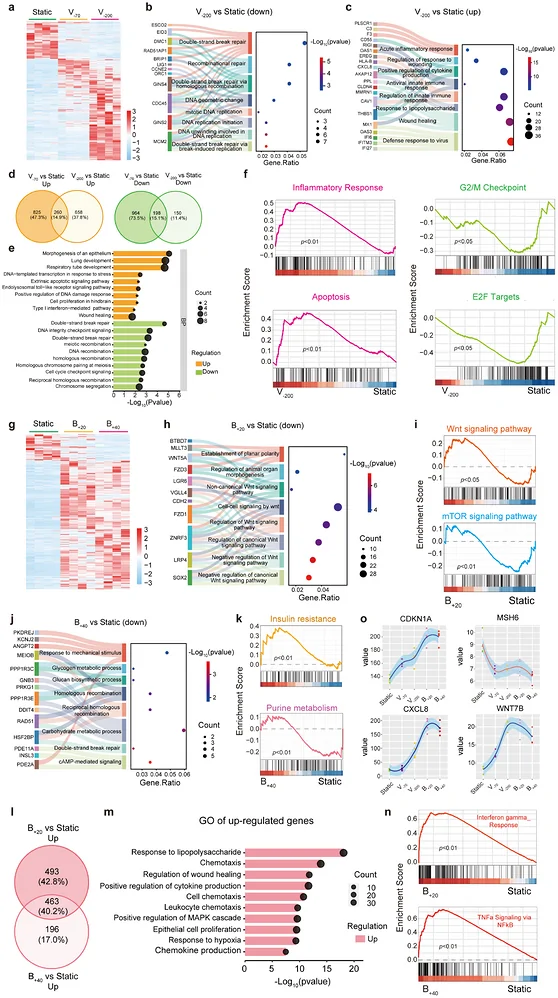
RNA‐seq analysis of OPECs and PMECs subjected to volutrauma and barotrauma in an acinus‐on‐a‐chip model. (a) Heatmap showing gene expression patterns of volutrauma‐treated acinar chips exposed to ‐70 mbar (V_‐70_) and ‐200 mbar (V_‐200_) vs. static controls. (b,c) Gene Ontology (GO) enrichment analysis of downregulated (b) and upregulated (c) genes in OPECs at V_‐200_. (d) Venn diagram analysis identified overlap of differential genes (V_‐70_ and V_‐200_ vs. static controls). (e) Enriched GO terms of the co‐upregulated and co‐downregulated genes in V_‐70_ and V_‐200_ vs. static controls. (f) Gene Set Enrichment Analysis (GSEA) of the top Hallmark gene sets enriched with upregulated and downregulated genes in V_‐200_ and static controls. (g) Heatmap of differentially expressed genes in barotrauma‐treated acinus‐on‐a‐chip exposure to +20 mbar (B_+20_) or +40 mbar (B_+40_) pressure (2 h) vs. static controls. (h) GO analysis of downregulated genes in B_+20_ vs. static controls. (i) GSEA analysis identified the enriched signaling pathways of OPECs in B_+20_ and static controls. (j) Enriched GO terms of downregulated genes in B_+40_ vs. static controls. (k) GSEA revealing enrichment in insulin resistance and purine metabolism pathways in B_+40_. (l) Venn analysis of co‐upregulated genes in B_+20_ and B_+40_ vs. static controls. (m) GO terms enriched among the co‐upregulated gene set common to both pressure conditions. (n) GSEA showing significant enrichment of interferon‐γ response and TNF‐α signaling in barotrauma‐upregulated genes(. o) Dynamic expression profiles of selected genes involved in DNA damage response, repair, inflammation, and Wnt signaling across different volutrauma‐ and barotrauma‐treated conditions. n = 4 biological replicates.

We further examined barotrauma effects on barrier integrity and cellular functions in OPECs and PMECs. Heatmap analysis revealed significant transcriptomic changes following barotrauma (Figure 4g and Figure S4e). GO analysis of downregulated genes in OPECs at B_+20_ (vs. static conditions) revealed significant enrichment in planar cell polarity, organ morphogenesis, and Wnt signaling regulation (Figure 4h). GSEA analysis of the upregulated genes further showed enrichment in gene sets related to the Wnt signaling pathway, while the downregulated genes were linked to the mTOR signaling pathway (Figure 4i). B_+40_ downregulated mechanosensory and glycogen/glucan metabolic genes while promoting insulin resistance and suppressing purine metabolism, revealing pressure‐specific dual disruption of structural and metabolic homeostasis (Figure 4j,k). Furthermore, comparative transcriptomics identified 463 genes commonly upregulated in the acinus‐on‐a‐chip at both B_+20_ and B_+40_, with functional annotation highlighting their involvement in chemotaxis, wound healing, and cytokine production (Figure 4l,m). GSEA analysis revealed that both B_+20_ and B_+40_ significantly enhanced inflammatory responses, particularly activating interferon‐γ response and TNF‐α signaling pathways (Figure 4n). Genes regulating DNA damage (*CDKNA1*, *MSH6*), apoptosis (*CXCL8*), and Wnt signaling (*WNT7B*) showed distinct expression patterns across volutrauma, barotrauma, and static conditions, correlating with observed epithelial dysfunction (Figure 4o). Transcriptomic profiling of B_+40_‐exposed PMECs revealed upregulated genes enriched in cell‐cell junction assembly and epidermal differentiation pathways, while downregulated genes predominantly involved reactive oxygen species metabolism and axonemal dynein complex assembly (Figure S4f, g). GSEA demonstrated significant enrichment of TNF signaling and cell adhesion molecule pathways, indicating establishment of a robust inflammatory microenvironment in barotrauma‐challenged endothelium (Figure S4h). Taken together, these findings indicate that both volutrauma and barotrauma can cause inflammatory response, apoptosis and injury of the acinar‐capillary barrier in the acinar chips, however their specific molecular mechanisms may exit difference: overexpansion of acinus caused by volutrauma mainly initiate epithelial cells’ DNA damage, however barotrauma with lower positive pressure of +20 mbar mainly cause epithelium damage by affecting the Wnt signaling pathway, but the injury caused by higher positive pressure of +40 mbar is mainly attributed to the dysfunction of cellular metabolism.

### 2.5 Volutrauma Elicits DNA Damage Responses (DDR), and Barotrauma Dysregulates Wnt Signaling and Cellular Metabolic Homeostasis

Given the transcriptional reprogramming induced by volutrauma and barotrauma, particularly in pathways governing DNA damage, apoptosis, inflammation, metabolism, and Wnt signaling pathways, we systematically analyzed the dysregulation of key genes within these pathways. RT‐qPCR revealed suppressed DNA damage repair in the acinus‐on‐a‐chip under both volutrauma (V_‐70_ and V_‐200_ mbar) and barotrauma (B_+20_ and B_+40_), with critical repair genes (*PCNA*, *PMS2*, *DDB2*) showing markedly reduced expression (Figure 5a). In addition, B_+40_ significantly downregulated OPA1 (31% reduction, *p* < 0.01) and PINK1 (27% reduction, *p* < 0.001), whereas B_+20_, V_‐70_, and V_‐200_ had no significant effect on these genes (Figure 5b), implying high‐pressure barotrauma specifically disrupts mitochondrial functions.

**FIGURE 5:**
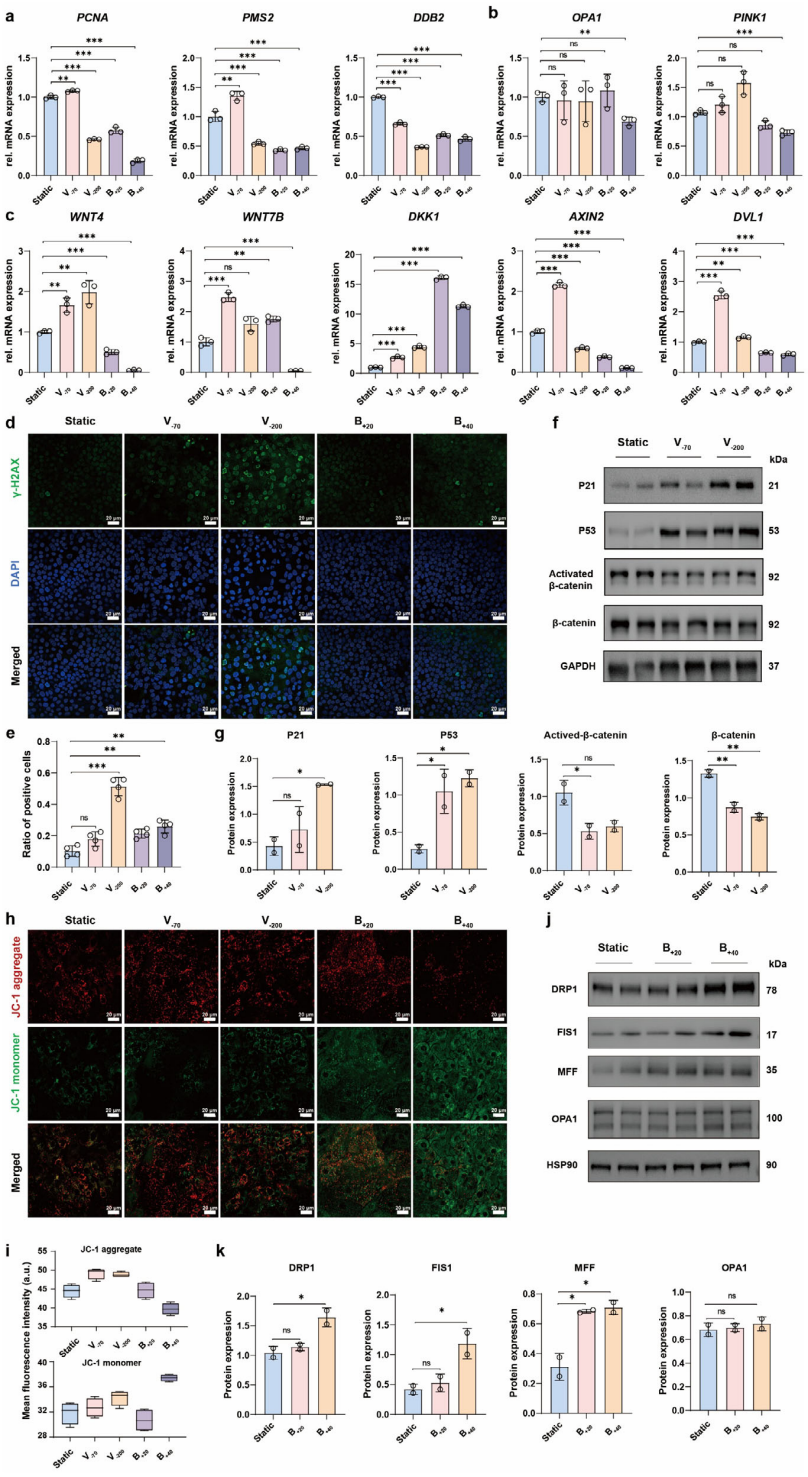
Progressive DNA damage response, Wnt signaling dysregulation, and mitochondrial homeostasis impairment underlie VILI susceptibility in the acinus‐on‐a‐chip. (a) RT‐qPCR analysis of mRNA expression levels of DNA repair genes (*PCNA*, *PMS2*, *DDB2*) in OPECs under static controls, volutrauma (V_‐70_ and V_‐200_), and barotrauma (B_+20_ and B_+40_). (b) mRNA levels of mitochondrial homeostasis regulators (*OPA1*, *PINK1*) at static, volutrauma, and barotrauma conditions. (c) Expression profiles of Wnt signaling components (*WNT4*, *WNT7B*, *AXIN2*, *DKK1*, *DVL1*) at static, volutrauma, and barotrauma conditions. (d,e) Immunofluorescence analysis of γ‐H2AX foci (DNA damage marker) in OPECs at static, volutrauma, and barotrauma (d), with quantification of γ‐H2AX positive (γ‐H2AX^+^) cells proportions (e). Scale bars: 20 µm. (f,g) Immunoblotting of DNA damage markers (P21, P53) and Wnt signaling effectors (activated β‐catenin, total β‐catenin) in static and volutrauma‐treated acinus‐on‐a‐chip models. GAPDH served as a loading control (f), with quantification of indicated proteins (g). (h,i) Immunofluorescence analysis of mitochondrial membrane potential (JC‐1 monomer/green vs. aggregate/red) in OPECs at static, volutrauma, and barotrauma (h), with mean fluorescence intensity quantification (i). Scale bars: 20 µm. (j,k) Immunoblotting analysis of mitochondrial fission regulators (DRP1, FIS1, MFF) and homeostasis marker (OPA1) in OPECs at static and barotrauma. HSP90 served as a loading control. (j), with quantification of indicated proteins (k). Data were presented as mean ± S.D., n = 3 biological replicates. Data were from one of three independent experiments. The data were analyzed by one‐way ANOVA with Tukey's multiple comparisons test (a, b, c, e, g, k). ^*^ *p* < 0.05, ^**^ *p* < 0.01, ^***^ *p* < 0.001, ns, not significant.

While Wnt signaling is well‐documented to regulate cell proliferation, differentiation, and apoptosis, its specific role in VILI pathogenesis remains elusive. Transcriptomic profiling previously identified Wnt signaling dysregulation in barotrauma‐treated acinus‐on‐a‐chip. Targeted gene analysis revealed Wnt ligands (*WNT4*/*7B*) were upregulated at volutrauma but suppressed under barotrauma, with the Wnt negative regulator *DKK1* showing stronger upregulation at barotrauma (Figure 5c). Conversely, Wnt transduction regulators *AXIN2* and *DVL1* exhibited opposing expression patterns, showing upregulated at V_‐70_ and V_‐200_ but downregulated at B_+20_ and B_+40_ (Figure 5c). These differential expression patterns highlight distinct modulatory effects of volutrauma and barotrauma on mitochondrial functions and Wnt signaling.

To validate DNA damage, we further quantified γ‐H2AX foci in OPECs under volutrauma/barotrauma. Pressure‐dependent accumulation of γ‐H2AX^+^ cells was observed, with V_‐200_ inducing the most severe DNA damage (> 50% γ‐H2AX^+^ cells, *p* < 0.001 vs. static) (Figure 5d, e). Barotrauma showed graded effects (B_+20_: 22%, *p* < 0.01; B_+40_: 25%, *p* < 0.001), while V_‐70_ remained indistinguishable from controls (< 20%, ns), indicating a mechanical threshold for genotoxic stress (Figure 5d,e). Immunoblotting further confirmed pressure‐dependent upregulation of P21 and P53 at V_‐70_ and V_‐200_ alongside modest β‐catenin reduction in Wnt signaling, revealing volutrauma‐induced DNA damage (Figure 5f,g). JC‐1 staining showed that B+40 induced significant membrane potential depolarization, with a 10.6% reduction in aggregate (red) fluorescence (44.4 to 39.7) and a 17.4% increase in monomer (green) intensity (31.8 to 37.4) (Figure 5h,i). Mitochondrial fission mediators (DRP1, FIS1, MFF) were progressively upregulated at B_+20_ to B_+40_ (Figure 5j,k), indicating high‐pressure barotrauma disrupts mitochondrial energetics and triggers early apoptotic signaling during acute VILI. Collectively, these results align with transcriptomic findings demonstrating that VILI pathogenesis involves distinct molecular mechanisms, where volutrauma may primarily drive cellular DNA damage, and barotrauma appears to dysregulate Wnt signaling and mitochondrial metabolism. These differential molecular signatures underscore the necessity for injury modality‐specific therapeutic strategies in mechanical ventilation management.

### 2.6 Single‐Cell RNA Sequencing Delineates Cellular Landscapes of VILI in the Acinus‐on‐a‐Chip

To characterize how volutrauma and barotrauma affect pulmonary epithelial differentiation, we performed single‐cell RNA sequencing (scRNA‐seq) on 38,511 cells of the acinus‐on‐a‐chip under three conditions: static, V_‐200_, and B_+40_ (Figure 6a and Figure S5a). Uniform Manifold Approximation and Projection (UMAP) analysis identified two major cell clusters: EPCAM^+^CDH1^+^ epithelial cells and PECAM1^+^CDH5^+^ endothelial cells (Figure S5b). We re‐clustered the epithelial cells and identified ten distinct subtypes derived from EPCAM^+^ cells, including basal cells (quiescent basal, basal S100A2^+^KI67^+^, basal P63^+^, and proliferating basal cells), secretory cells (cell cycling secretory and mature secretory cells), and four intermediate clusters (Basal/Sec Trans_a/b/c, and Sec/Alveolar Trans cells), each characterized by distinct molecular signatures (Figure 6b,c and Figure S5c). Volcano plots illustrated significantly differentially expressed genes among these lineages (Figure 6d). GO analysis revealed that the quiescent basal and basal P63^+^ clusters were highly enriched in the Wnt signaling pathway. The clusters of Basal/Sec Trans_a, basal S100A2^+^KI67^+^, cell cycling secretory, and proliferating basal mainly exhibited a similar signature response related to DNA replication and RNA splicing. The clusters of Basal/Sec Trans_b and mature secretory were strikingly enriched for responses related to cellular metabolism, including oxidative phosphorylation and cellular respiration (Figure 6e).

**FIGURE 6:**
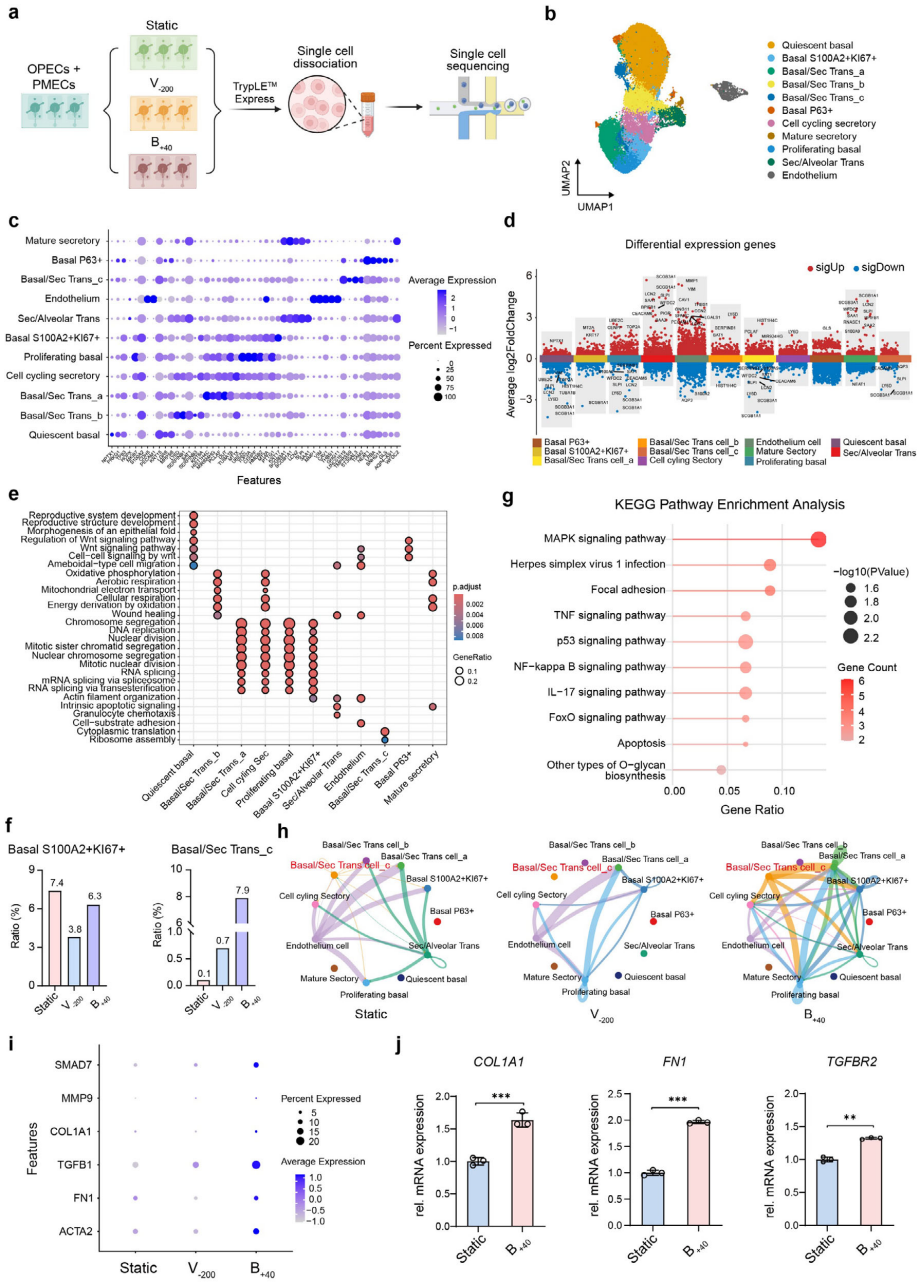
scRNA sequencing reveals distinct OPECs landscapes in the acinus‐on‐a‐chip model under volutrauma and barotrauma. (a) Schematic workflow for single‐cell RNA sequencing (scRNA‐seq) analysis of OPECs in the acinus‐on‐a‐chip before (static) and after the indicated VILI treatment: ‐200 mbar volutrauma (V_‐200_) and +40 mbar barotrauma (V_+40_). (b) UMAP plot of single cells profiled OPECs in the acinus‐on‐a‐chip before and after VILI. (c) Bubble plot of the average and percent expression of different markers in each cell type across the indicated cell clusters. (d) Differential gene expression analysis revealed upregulated and downregulated genes across various cell types. (e) GO enrichment analysis on differentially expressed genes in each cell cluster. (f) Cell proportions analysis of the cell clusters in Basal S100A2^+^KI67^+^ and Basal/Sec Trans_c at static, V_‐200_, and B_+40_. (g) KEGG enrichment analysis of the differentially expressed genes in the cell cluster of Basal/Sec Trans_c at B_+40_ vs. static controls. (h) Network of cell‐cell communication showing different numbers of interactions in TGF‐β signaling at static, V_‐200_, and B_+40_. (i) Dot plot visualization of differentially expressed fibrotic marker genes across Basal/Sec Trans_c in OPECs at static, V_‐200,_ and B_+40_, with dot size reflecting expression frequency and color intensity indicating normalized expression levels (log_2_TPM), highlighting significant upregulation of pro‐fibrotic transcripts in barotrauma conditions. (j) RT‐qPCR analysis of the indicated fibrotic signature genes in OPECs subjected to static and B_+40_ treatments within acinus‐on‐a‐chip. Data were presented as mean ± S.D., n = 3 biological replicates. Data were from one of three independent experiments. The data were analyzed by two‐tailed Student's *t*‐test (j), ^**^ *p* < 0.01, ^***^ *p* < 0.001.

Moreover, we assessed ratio differences of the indicated epithelial cell subtypes at static, V_‐200_, and B_+40_. Figure 6f and Figure S5d,e indicated a slight decline in the proportions of basal S100A2^+^KI67^+^, proliferating basal, Basal/Sec Trans_a, and cell cycling secretory cells at V_‐200_ and B_+40_, suggesting that both volutrauma and barotrauma impair the proliferative programming of pulmonary progenitor cells. V_‐200_ expanded quiescent basal cells (45.4% to 53.5%) and Basal/Sec Trans_b populations (10.2% to 12.1%), while B_+40_ specifically drove a 79‐fold Basal/Sec Trans_c expansion (0.1% to 7.9%). These distinct response patterns reveal injury specificity of volutrauma and barotrauma in pulmonary acinar epithelial differentiation. KEGG pathway analysis of barotrauma‐induced Basal/Sec Trans_c cells revealed significant enrichment in injury‐response pathways related to the MAPK signaling pathway, P53 signaling pathway, apoptosis, and activation of immune responses (Figure 6g). GO enrichment analysis mainly identified multiple metabolic processes enriched in Basal/Sec Trans_c lineage (Figure S5f), indicating VILI‐engaged acinar epithelial cells exhibited aberrant metabolic reprogramming, especially under barotrauma. CellChat analysis revealed barotrauma‐specific TGF‐β hyperactivation vs. volutrauma's mild suppression, implying that barotrauma preferentially activates fibrotic remodeling (Figure 6h and Figure S5g). To validate barotrauma's impact on epithelial fibrotic progression, we analyzed fibrotic regulators in single‐cell RNA sequencing data. Figure 6i indicated that B_+40_ significantly upregulated key fibrotic genes, such as *TGFB1*, *ACTA2*, *FN1*, and elevated COL1A1 protein in TGF‐β1‐treated OPECs (Figure S5h). RT‐qPCR and immunofluorescence staining confirmed barotrauma‐induced overexpression of profibrotic markers (*COL1A1*, *FN1*, *TGFβR2*) and P63^+^COL1A1^+^ fibrotic cells in the acinus‐on‐a‐chip (Figure 6j and Figure S6). Consistently, in TGF‐β1‐primed OPECs, barotrauma amplified expression of *COL1A1*, *ACTA2*, and *TGFβ1*, demonstrating its synergistic potentiation of fibrotic pathways (Figure S5i). Collectively, these findings demonstrate that volutrauma and barotrauma disrupt OPECs homeostasis and compromise lineage integrity. Critically, we establish that barotrauma exacerbates fibrotic progression through TGF‐β‐driven ECM remodeling and potentiates clinical VILI complications including ventilator‐associated fibrosis and acute respiratory deterioration.

### 2.7 Combined DDR/Wnt/Mitochondrial Modulation Mitigates Volutrauma and Barotrauma in Acinus‐on‐a‐Chip

To identify actionable therapeutic strategies for VILI, we systematically interrogated three key pathological pathways implicated in its pathogenesis: DNA damage response (DDR), Wnt/β‐catenin signaling, and mitochondrial homeostasis. For volutrauma, which primarily involves DDR activation, we evaluated pharmacological inhibition of P21, a key mediator of DDR‐driven apoptosis, as a potential intervention. Figure 7a indicated cyclic mechanical stretch (V_‐200_, 0.2 Hz) induced robust upregulation of DDR markers (P21: 1.5‐fold increase, *p* < 0.05; P53: 3.1‐fold increase, *p* < 0.01) and downstream inflammatory/apoptotic responses (p‐P65: 5.8‐fold increase, *p* < 0.001; cleaved caspase‐3: 1.2‐fold increase). Treatment with the P21 inhibitor UC2288 (5–20 µM) during V_‐200_ dose‐dependently reversed these effects: at 15 µM, P21 levels decreased to 9.0‐fold (*p* < 0.001), P53 to 2.8‐fold (*p* < 0.001), p‐P65 to 4.3‐fold (*p* < 0.001), and cleaved caspase‐3 to 1.5‐fold (*p* < 0.05) (Figure 7b). Multiplex ELISA confirmed that this DDR blockade significantly attenuated NF‐κB‐dependent cytokine storms, with IL‐6 (455 to 374 pg/mL, *p* < 0.001), IL‐8 (10,175 to 7,553 pg/mL, *p* < 0.01), and TNF‐α (23 to 13 pg/mL, *p* < 0.01) all significantly reduced (Figure 7c). These findings indicate that DDR inhibition simultaneously mitigates DNA damage, inflammation, and apoptotic cell death caused by volutrauma.

**FIGURE 7:**
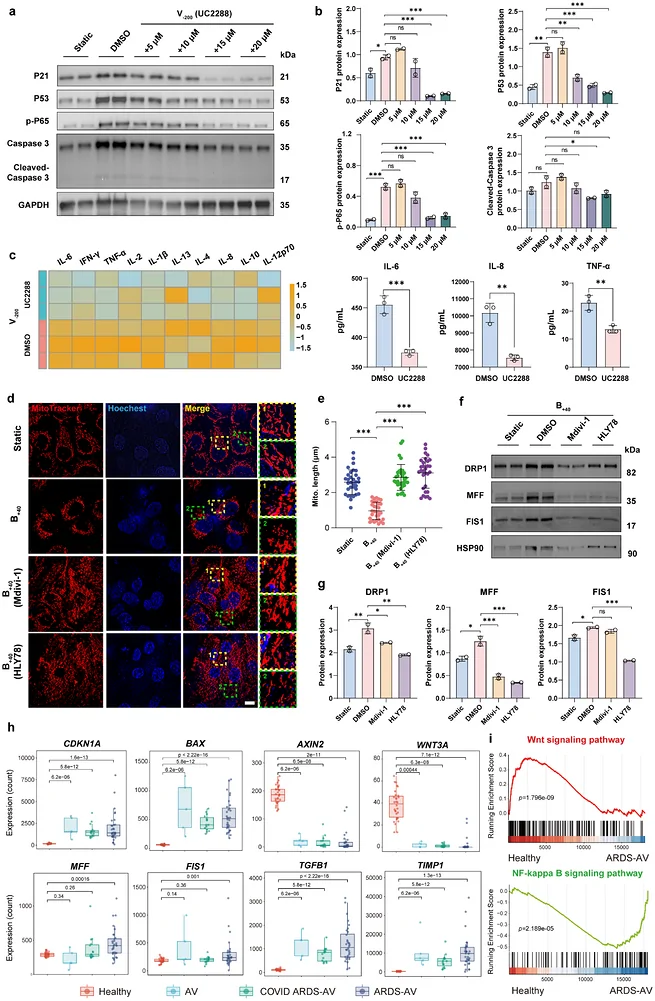
Identification of DDR pathway modulation, Wnt signaling manipulation, and mitochondrial dynamics regulation as therapeutic targets for VILI pathogenesis. (a,b) Dose‐dependent suppression of DDR‐associated pro‐apoptotic proteins by UC2288 treatment in V_‐200_‐exposed acinus‐on‐a‐chip (a). Quantified protein expression levels of P21, P53, phosphorylated P65 (p‐P65), and cleaved‐caspase3 demonstrate concentration‐dependent attenuation following UC2288 pretreatment (b). (c) UC2288 pretreatment attenuated inflammatory cytokine profiles in V_‐200_‐treated acinus‐on‐a‐chip. Secretion levels of IL‐6, TNF‐α, and IL‐8, etc., were quantified by multiplex immunoassay. (d,e) Super‑resolution imaging of mitochondrial morphology in OPECs stained with MitoTracker Red CMXRos, under conditions with and without pharmacological intervention (Mdivi‐1: DRP1 inhibitor, 10 µM; HLY78: Wnt activator, 10 µM) (d), and corresponding quantitative analysis of mitochondrial length (e). Scale bar: 10 µm. (f,g) Pretreatment with Mdivi‐1 and HLY78 in B_+40_‐treated acinus‐on‐a‐chip decreased the protein expression of the indicated proteins related to mitochondrial fission (f), and the corresponding quantitative analysis (g). (h) Box plots of mRNA levels of indicated genes in healthy, assisted ventilation (AV), COVID ARDS‐AV, and ARDS‐AV specimens (using datasets GSE163426 and GSE237252). In the box plots, the middle line depicts the median and the whiskers in the min‐to‐max range. (i) GSEA analysis of the enriched signaling pathways in healthy individuals and ARDS‐AV patients. Data were presented as mean ± S.D., n = 3 biological replicates. Data were from one of three independent experiments. The data were analyzed by two‐tailed Student's t‐test (c) and one‐way ANOVA with Tukey's multiple comparisons test (b, e, g, h). ^*^ *p* < 0.05, ^**^ *p* < 0.01, ^***^ *p* < 0.001, ns, not significant.

Integrating transcriptomic and scRNA‐seq data revealing barotrauma‐induced Wnt/metabolic dysregulation, we further employed MitoTracker to quantify mitochondrial structural damage. Super‐resolution imaging via structured illumination microscopy (SIM) revealed that barotrauma (B_+40_) induced severe mitochondrial fragmentation (average length reduced 0.6‐fold vs. static controls, *p* < 0.001), and was rescued by Mdivi‐1 (a DRP1 inhibitor) or HLY78 (a Wnt activator) (Figure 7d,e). Immunoblotting analysis confirmed that fission inhibition by Mdivi‐1 and Wnt activation by HLY78 could significantly downregulate DRP1/MFF/FIS1 protein levels to preserve mitochondrial functions, respectively (Figure 7f,g), consisting with the barrier permeability assays (Figure S7). These results nominate mitochondrial fission inhibition and Wnt pathway activation as potential rescue strategies for barotrauma. Analysis of Gene Expression Omnibus datasets (GSE163426 and GSE237252) further confirmed strong clinical concordance between in vitro acinus‐on‐a‐chip modeling of volutrauma/barotrauma‐induced pathway dysregulation and human ARDS pathophysiology. Figure 7h and Figure S8a demonstrated consistent findings of acinus‐on‐a‐chip with dysregulated DNA damage response (*CDKN1A*, *BAX*, *H2AX*, *GADD45A*), Wnt signaling (*CTNNB1*, *FZD1*, *AXIN2*, WNT3A), mitochondrial dynamics (*MFF*, *FIS1*, *MFN1*, *OPA1*), and fibrotic remodeling (*TGFB1*, *TIMP1*, *MMP7*, *MMP9*) in ARDS cohorts (including COVID‐associated) compared to healthy controls. GSEA further identified downregulated Wnt/mTOR transcriptional signatures and upregulated P53, NF‐κB, TNF, and apoptotic pathways in ARDS patients, establishing mechanistic parallels between chip‐modeled injury and clinical VILI pathogenesis (Figure 7i and Figure S8b). Therefore, our integrated findings revealed divergent mechanisms for these mechanical injuries of VILI: volutrauma primarily induced DDR activation, driving P53‐mediated apoptosis and subsequent NF‐κB‐dependent inflammatory cytokine storm, while barotrauma disrupts mitochondrial dynamics through DRP1 overactivation and simultaneously suppresses canonical Wnt signaling. This dual disruption creates a feedforward loop of metabolic‐energetic failure, exacerbating cellular injury.

In summary, by mimicking the spatial architecture, fluid dynamics, and multicellular lineages of human pulmonary acini through microfabrication and organoid technology, we developed an acinus‐on‐a‐chip platform as a highly pathophysiologically relevant model to decouple the pathogenic mechanism of volutrauma and barotrauma, and identified potential therapeutic targets. As illustrated in Figure 8, volutrauma primarily arises from DNA damage response (DDR) activation, which sequentially induces P53‐mediated apoptosis and NF‐κB‐driven inflammatory amplification, whereas barotrauma stems from mitochondrial fission dysregulation coupled with concurrent Wnt pathway suppression, leading to metabolic collapse. The sustained inhibition of canonical Wnt signaling observed in barotrauma exacerbates mitochondrial dysfunction, creating a feedforward loop of metabolic‐energetic failure. Notably, barotrauma specifically exacerbates a fibrotic remodeling of the epithelial cells. These mechanistic insights delineate targeted therapeutic strategies: pharmacological inhibition of P21 effectively disrupts the DDR‐apoptosis‐inflammation axis in volutrauma, while combined modulation of mitochondrial fission dynamics through DRP1 inhibition and Wnt pathway reactivation via β‐catenin stabilization restores mitochondrial integrity and metabolic homeostasis in barotrauma. Collectively, this study offers a potential framework for developing differential intervention strategies in ARDS, suggesting that the acinar chip may be capable of resolving clinically relevant ventilation‑associated pathologies through pathway‑specific modulation.

**FIGURE 8:**
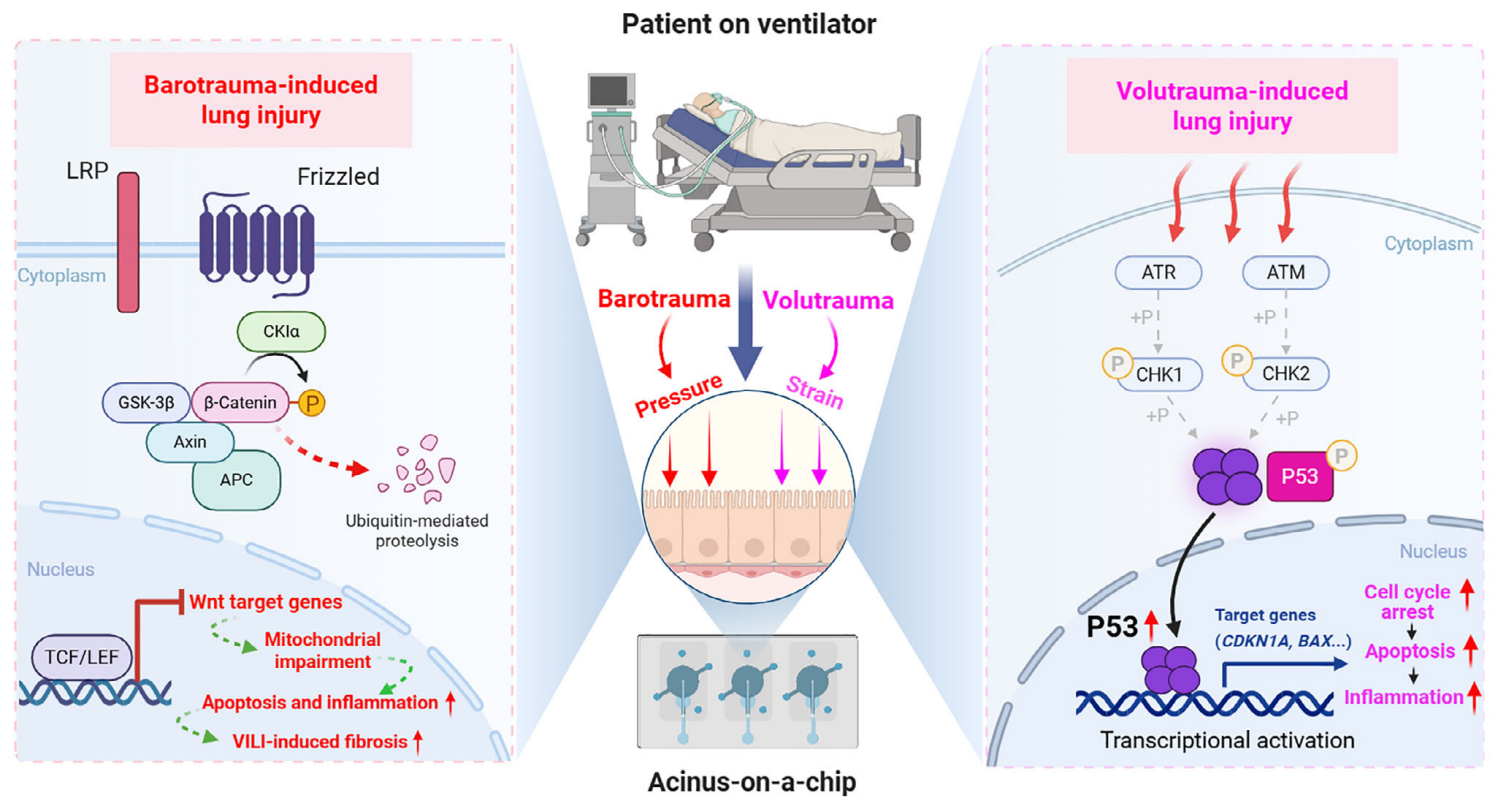
Schematic representation of ex vivo VILI pathomechanism interrogation using the acinus‐on‐a‐chip platform. The acinus‐on‐a‐chip platform recapitulates key pathophysiological features of volutrauma (excessive strain) and barotrauma (high pressure) by independently modulating mechanical forces during ventilation, thereby enabling mechanistic interrogation of DNA damage response (DDR) signaling, mitochondrial homeostasis, and Wnt/β‐catenin pathway crosstalk.

## 3 Conclusion

Overall, our human acinus‐on‐a‐chip VILI model offers advantages over current in vitro systems by recapitulating disease‐relevant biomechanical and pathophysiological hallmarks, most notably the self‐sustaining inflammatory cascade central to clinical VILI progression. This platform applies distinct pressure modalities to separately simulate the key pathological features of volutrauma and barotrauma, offering a potential tool for investigating the mechanical dynamic crises associated with mechanical ventilation in intensive care settings. However, current limitations include the absence of neutrophils, key mediators of early injury amplification, and fibroblasts, critical to fibrotic remodeling. Of note, this exclusion is mitigated by the chip's modular architecture, which permits iterative incorporation of immune and stromal compartments as demonstrated in prior multilineage lung‐on‐chip iterations [28, 29, 30]. Meanwhile, while our platform enables mechanistic interrogation of air‐blood barrier mechanobiology, its fidelity is constrained by inherent limitations of polydimethylsiloxane (PDMS) membranes [12, 31, 32, 33, 34]. Specifically, PDMS is known to absorb hydrophobic small molecules, including many drugs and signaling lipids, leading to reduced effective concentrations and altered dose‐response curves in microfluidic systems [33, 34]. This absorption could underestimate therapeutic efficacy or mask off‐target effects in our pharmacological interventions, requiring careful validation with PDMS‐free controls or mass balance analysis. Moreover, the mechanical mismatch between PDMS (elastic modulus ∼1‐2 MPa) and native lung parenchyma (∼0.1‐0.5 kPa for alveolar septa) introduces a non‐physiological substrate stiffness that may aberrantly activate mechanosensitive pathways such as YAP/TAZ, integrin‐mediated focal adhesion signaling, and cytoskeletal remodeling [35, 36]. This altered mechanical microenvironment could confound interpretation of stretch‐induced mechanotransduction cascades, including the DDR‐P53 and Wnt pathways central to our study, by superimposing stiffness‐driven signals onto pressure‐driven ones. To mitigate these limitations, future iterations of the acinus‐on‐a‐chip should employ surface‐modified PDMS such as parylene‐C coating or PEG grafting to reduce drug absorption while preserving optical transparency and gas permeability [37, 38]. Alternatively, replacing PDMS with low‐absorption elastomers such as polyurethane or polydimethylsiloxane‐polyethylene glycol block copolymers, or incorporating hydrogel‐based membrane layers with tunable stiffness matching native alveolar tissue such as gelatin methacryloyl or collagen‐elastin composites, would simultaneously address both absorption and mechanical mismatch [39, 40].

In addition, the acinus‐on‐a‐chip provides a potential platform for translation from fundamental mechanobiology research to clinical applications. First, the platform could support high‐throughput screening of anti‐VILI drug candidates targeting specific molecular nodes, including P21 inhibitors, Wnt agonists, and mitochondrial fission blockers, in human tissues with patient‐specific genetic backgrounds [41, 42]. Second, the chip's precise control of pressure waveforms enables simulation of various clinical ventilation modes, facilitating systematic assessment of patient‐specific organoid responses to guide personalized optimization of ventilator parameters, including frequency, tidal volume, positive end‐expiratory pressure (PEEP), and driving pressure, based on each patient's pulmonary mechanical sensitivity [43, 44]. Third, integration with multi‐omics readouts may identify biomarkers predictive of VILI susceptibility, enabling risk stratification in intensive care settings. Fourth, future efforts could focus on expanding the platform to support ARDS patient cohorts by incorporating patient‐derived immune cells (like peripheral blood mononuclear cells, PBMCs), thereby establishing patient‐specific VILI models for rapid screening and personalized evaluation of clinical agents such as dexamethasone, ultimately providing guidance for clinical pharmacotherapy [45, 46, 47].

In conclusion, our acinus‐on‐a‐chip platform successfully recapitulates key pathophysiological hallmarks of clinical VILI, including acinar‐capillary barrier dysfunction and mechano‐inflammatory crosstalk that remain unmodeled in conventional static cultures. This physiological fidelity establishes the system as a transformative preclinical model for mechanistic discovery and therapeutic development, enabling simultaneous identification of disease biomarkers, mechanotransductive pathways, and druggable targets specific to VILI progression.

## 4 Experimental Methods

### 4.1 Ethics Approval

Human lung clinical specimens were obtained from the First Affiliated Hospital of Guangzhou Medical University. Ethical approval for the use of these specimens was granted by the Ethics Committee of the First Affiliated Hospital of Guangzhou Medical University (Approval No. ES‐2024‐095‐04).

### 4.2 Device Fabrication and Characterization

To establish a biomimetic model of pulmonary acinar architecture, we developed a seven‐layered microfluidic platform through precision stereolithographic molding of PDMS prepolymer (10:1 w/w base to curing agent, Dow Corning, USA). Sequential 80 °C curing steps enabled the obtaining of the hierarchical structures comprising terminal bronchiole, respiratory bronchiole, alveolar duct/sac, alveolus, vascular chamber, and pneumatic unit layers. The integrated device features five distinct microstructural components: (i) a length 5.0 mm, width and height 1.0 mm top microchannel for air flow, (ii) a 2.5 mm radius × 1.5 mm height cylindrical transition element, (iii) a conical alveolar duct with 2.5 mm apical and 1.0 mm basal radii, (iv) a 14 × 6 × 1 mm rectangular perfusion channel, and (v) a 1.5 mm radius × 1.5 mm height basal reservoir chamber. The 10 µm‐thick microporous PDMS membranes were fabricated by pressure casting on silicon templates with 7 µm‐diameter posts. The 20 µm‐thick nonporous PDMS membranes were spin‐coated on a blank silicon wafer (1000 rpm for 30 s, 2000 rpm for 60 s). All layers were plasma activated (Electro‐Technic Products) and precisely aligned layer‐by‐layer to ensure robust, leak‐free bonding of the complete acinus‐on‐a‐chip device. The pressure levels and frequency (0.2 Hz) employed in our acinar chip were selected based on the strain, transpulmonary pressure, and frequency of pulmonary acini under physiological respiration and clinical mechanical ventilation conditions [19, 42]. The negative pressure values (−70, −100, and −200 mbar) were selected based on the strain of the acinus under both physiological respiration (−70 mbar, 8% strain), the threshold of injury (−100 mbar, 12% strain), and pathological (volutrauma) conditions (−200 mbar, 18% strain). The positive pressure values (+20, +30, and +40 mbar) were selected to simulate barotrauma from elevated transpulmonary pressures during mechanical ventilation, spanning from near the safety threshold (19.6∼24.5 mbar [27]) to supraphysiological levels that induce progressively severe injury.

### 4.3 Simulation of Gas Flow Dynamics

Gas flow simulations in two distinct PDMS microchambers was performed using COMSOL Multiphysics(Burlington, MA): (1) the acinus‐on‐a‐chip featuring an L‐shaped channel (inlet width = 1 mm, length = 5 mm, height = 1 mm) connected to an inverted trapezoidal chamber (bottom width = 2 mm, top width = 5 mm, height = 2 mm); and (2) the reported chip [17] comprising a square chamber (side length = 4.5 mm). Air was injected at the inlet under a pressure boundary condition of 2000 Pa, with the outlet set to atmospheric pressure. The simulations assumed laminar, compressible flow, and all walls were assigned no‐slip boundary conditions to model realistic gas‐surface interactions.

### 4.4 Mechanical Characterization

Following chip assembly, the mechanical properties of the microfluidic device were evaluated using a 3D Surface Profiler (VK‐X3000, KEYENCE). To quantify the deformation under mechanical stress, the assembled chips were subjected to controlled air pressure within a custom loading apparatus. Pressure stimuli were applied incrementally, ranging from 0 to 1000 mbar (positive pressure) and ‐800 to 0 mbar (negative pressure), with image acquisition performed at each interval to record real‐time topographical changes. The system's non‐contact laser scanning captured high‐resolution surface profiles, allowing for precise calculation of the maximum vertical displacement (Δh) of the flexible membrane. Stress‐strain characteristics were subsequently derived by correlating the applied pressures with the measured morphometric data, thereby validating the chip's capacity to deliver physiologically relevant mechanical strain.

### 4.5 Lung Tissue Preparation

Freshly resected distal lung tissue (∼1 cm^3^) was obtained from normal regions adjacent to lung adenocarcinoma lesions [48, 49, 50]. Prior to organoid derivation, we performed H&E staining on each tissue fragment to confirm histological normality. Immunofluorescence staining and H&E staining indicated that, compared with matched tumor tissue, the selected paracancerous tissue exhibited intact acinar architecture, organized alveolar epithelial cell distribution, normal expression patterns of alveolar markers such as pro‐SPC and RAGE, and no evidence of tumor cell infiltration or architectural distortion (Figure S9). Only tissues meeting these histopathological criteria were used for subsequent experiments of organoid derivation and expansion.

### 4.6 Culture of Lung Acinar Organoids

Tissues were immediately immersed in ice‑cold Hank's Balanced Salt Solution (HBSS) supplemented with antibiotics (200 U/mL penicillin, 200 µg/mL streptomycin, 0.5 µg/mL amphotericin B) within 1 h of surgical excision. The tissue was rinsed three times with ice‐cold HBSS, minced into ≤1 mm^3^ fragments using sterile surgical blades, and washed with 10 mL of cold basal medium (DMEM/F12). After centrifugation at 400 × g for 5 min at 4 °C, the pellet was resuspended in 8 mL of digestion medium (DMEM/F12 containing 0.001% DNase I, 1 mg/mL collagenase I, and 1% antibiotic‑antimycotic). Digestion proceeded at 37 °C in a shaking incubator (120 rpm) for ∼60 min, with mechanical trituration every 10 min using a 10 mL serological pipette. Then, the digest was passed through a 70 µm cell strainer, and fetal bovine serum (FBS) was added to the filtrate (final concentration 2%) to halt enzymatic activity. Following centrifugation at 400 × g for 5 min at 4 °C, the cell pellet was treated with 2 mL of erythrocyte lysis buffer for 5 min at room temperature, then washed with cold basal medium. After another centrifugation, the pellet was resuspended in ice‐cold growth factor‐reduced Matrigel (80–160 µL per tissue fragment). 20 µL drops of the Matrigel‐cell suspension were dispensed into each well of a pre‐warmed 24‐well culture plate and allowed to solidify at 37 °C for 15–20 min. Subsequently, 500 µL of complete expansion medium (see Supplementary Table S1) was gently overlaid. Cultures were maintained at 37 °C under 5% CO_2_, with medium replacement every 5–6 days. Organoid formation was monitored by light microscopy. Additionally, organoids were passaged every 2–3 weeks. Matrigel droplets were disrupted by pipetting the medium ∼10 times, and the suspension was collected into a 15 mL tube. Cold basal medium was added to 10 mL, and the tube was incubated on ice for 5 min before centrifugation (400 × g, 5 min, 4 °C). The pellet was washed once with cold basal medium, then resuspended in 1 mL of TrypLE Express and incubated at 37 °C for 10–15 min. Digestion was terminated by adding 2% FBS. Organoids were mechanically dissociated into single cells and small clusters by pipetting 20–40 times with a Pasteur pipette. After washing and centrifugation, the cells were resuspended in an appropriate volume of ice‐cold Matrigel. 20 µL drops were plated, solidified, and cultured as described above.

### 4.7 Cell Culture in the Acinus‐on‐a‐Chip

For chip‐based culture, Matrigel‐embedded organoids were first treated with pre‐chilled Cell Recovery Solution (Corning, #354253) for 30 min on ice to dissolve the matrix. Liberated organoids were pelleted (500 × g, 3 min), enzymatically dissociated into single cells using TrypLE Express (Gibco, MA, USA; 37 °C, 15 min), and resuspended in serum‐free medium. Prior to cell seeding, the chip was sterilized by UV irradiation and ethanol washing, followed by coating both sides of the porous membranes with an extracellular matrix (ECM) mixture (150 µg/mL collagen I, 300 µg/mL fibronectin, and 60 µg/mL laminin in PBS) at 37 °C for 4–6 h. After removal of ECM, alveolar epithelial cells (Cell Biologics, #H‐6053, 5 × 10^5^ cells/mL) or single‐cell suspensions of OPECs (5 × 10^5^ cells/mL) were loaded into the acinus‐on‐a‐chip via pipette and cultured for 24 h. Following chip inversion, PMECs (ScienCell, #17821, 1 × 10^6^ cells/mL) were perfused to establish a confluent vascular layer. Following 3 days of culture, barrier integrity was confirmed through microscopy, tight junction immunofluorescence, and permeability assays, enabling mechanical stretch and ventilation studies.

### 4.8 Permeability Assay

Prior to permeability assessment, inlet and outlet reservoirs of the microfluidic device were evacuated. Cascade Blue (100 µg/mL; Life Technologies, Carlsbad, CA, USA, #C687) and 3 kDa Texas Red‐dextran (100 µg/mL; Life Technologies, #D3329) were reconstituted in flow medium (serum‐free DMEM/F12). A 500 µL aliquot of the dual‐tracer solution was loaded into the apical channel inlet reservoir, while 500 µL of tracer‐free flow medium was introduced to the basal channel inlet. Following tracer loading, both channels were subjected to a 5 min priming flush (600 µL/hour) to ensure homogeneous distribution throughout the podocyte chamber and channel network. Subsequently, both channels were perfused at 120 µL/hour for 2 h under physiological shear conditions (37 °C, 5% CO_2_). Effluent medium from outlet reservoirs was collected for fluorescence quantification (ex/em: 380/420 nm for Cascade Blue; 595/615 nm for Texas Red) using a multimode plate reader (BioTek Synergy H1). Apparent permeability coefficients (*P*_app_) were calculated as per established methods [51], accounting for tracer diffusion kinetics and chamber geometry.

### 4.9 Immunostaining

Following PBS washing (three times, 5 min each), cells were fixed with 4% paraformaldehyde (PFA) in PBS for 20 min at room temperature (RT). After three additional PBS washes, cells were permeabilized with 0.5% Triton X‐100 in PBS for 10 min. Non‐specific binding sites were blocked by incubating cells with 3% bovine serum albumin (BSA/PBS) for 1 h at RT. Primary antibodies (1:1000 dilution) were applied overnight at 4 °C. After PBS rinsing, cells were incubated with Alexa Fluor 488/594‐conjugated species‐matched secondary antibodies (1:500; Beyotime Biotechnology, Shanghai, China) for 1 h at RT. Nuclear counterstaining was achieved with 4′,6‐diamidino‐2‐phenylindole (DAPI; 1 µg/mL, Absin Bioscience Inc., Shanghai, China) for 10 min. Fluorescence imaging was performed using a Zeiss LSM 980 confocal microscope (Carl Zeiss AG, Jena, Germany) equipped with 405 nm (DAPI), 488 nm (Alexa Fluor 488), and 594 nm (Alexa Fluor 594) laser lines. Z‐stack images (0.5 µm optical sections) were acquired with Zen Blue software (v3.7) and processed using ImageJ (Fiji v2.14). Super‐resolution imaging was performed by structured illumination microscopy (SIM) (Carl Zeiss Elyra 7).

### 4.10 Immunoblotting

Cells in the acinar chips were lysed in RIPA buffer (Cell Signaling Technology, Danvers, MA, USA) supplemented with phosphatase inhibitor cocktail C (Santa Cruz Biotechnology, Dallas, TX, USA). Protein concentrations were quantified using the Enhanced BCA Protein Assay Kit (Pierce Biotechnology, Rockford, IL, USA), with 20 µg of total protein per lane resolved by SDS‐PAGE on 12% gels. Separated proteins were electrophoretically transferred to nitrocellulose membranes (0.45 µm pore size, Amersham, GE Healthcare Life Sciences, Pittsburgh, PA, USA). Membranes were blocked for 1 h at RT with 5% (w/v) non‐fat dry milk in Tris‐buffered saline with 0.1% Tween‐20 (TBST: 25 mM Tris‐HCl, 150 mM NaCl, 2 mM KCl, pH 7.4). Primary antibodies were diluted in blocking buffer and incubated overnight at 4 °C (Table S2). After three 10 min TBST washes, membranes were probed with HRP‐conjugated secondary antibodies (Table S2) for 1 h at RT. Following three additional 10 min TBST washes, protein bands were visualized using Amersham ECL Prime Western Blotting Detection Reagent (GE Healthcare Life Sciences) and imaged on a ChemiDoc Touch Imaging System (Bio‐Rad Laboratories, Hercules, CA, USA). This process was maintained for 1–2 days.

### 4.11 Cell Viability Assays

Cell viability was assessed using the Cell Counting Kit‐8 (CCK‐8, DOJINDO, CK04) following the manufacturer's instructions. Briefly, 10 µL of CCK‐8 reagent was added to each acinar chip, followed by incubation for 2 h at 37 °C. The absorbance of formazan products, proportional to cellular dehydrogenase activity, was measured at 450 nm using a microplate reader (BioTek Synergy H1, VT, USA), with a reference wavelength of 650 nm to correct for background interference. Viability was calculated as follows:

$$\begin{array}{ccc} \mathrm{Viability}\left(\%\right) & = & \left(A_{\mathrm{treatment}}-A_{\mathrm{blank}}\right)/\left(A_{\mathrm{control}}-A_{\mathrm{blank}}\right)\\ & & \times 100 \end{array}$$

where *A*_treatment_, *A_control_*, and *A_blank_* represent the absorbance of treated cells, untreated controls, and cell‐free medium, respectively. All experiments were performed in triplicate across three independent biological replicates.

### 4.12 Cell Cycle Analysis

OPECs cultured in acinar chips were subjected to volutrauma (12 h) and barotrauma (2 h). Following mechanical injury, cells were pulsed with 10 µM 5‐ethynyl‐2′‐deoxyuridine (EdU) in fresh culture medium for 2 h at 37 °C to label proliferating cells. Cells were enzymatically dissociated using a combination of Organoid Harvesting Solution (R&D Systems, Inc., Minneapolis, MN, USA) and TrypLE Express Enzyme (Thermo Fisher Scientific, Waltham, MA, USA), followed by neutralization with DMEM/F12 containing 10% FBS. Single‐cell suspensions were fixed in 4% paraformaldehyde (PFA/PBS) for 15 min at RT and permeabilized with 0.5% Triton X‐100 in PBS for 12 min. After blocking with 3% bovine serum albumin (BSA/PBS) for 30 min, EdU incorporation was detected using the BeyoClick EdU‐488 Cell Proliferation Kit (Beyotime Biotechnology, Shanghai, China) according to the manufacturer's protocol. Labeled cells were washed twice with PBS, resuspended in 500 µL of fluorescence‐activated cell sorting (FACS) buffer (PBS + 1% BSA + 2 mM EDTA), and analyzed on a BD FACS Aria II flow cytometer (BD Biosciences, San Jose, CA, USA) equipped with a 488 nm laser. Data were processed using FlowJo software (v10.8.1, BD Biosciences).

### 4.13 Mitochondrial Membrane Potential (ΔΨm) Measurement

Following volutrauma/barotrauma treatment on acinar chips for specified durations (volutrauma: 12 h; barotrauma: 2 h), mitochondrial depolarization was induced in positive controls by adding 10 µM carbonyl cyanide m‐chlorophenyl hydrazone (CCCP; Sigma‐Aldrich, St. Louis, MO, USA) to designated chips for 20 min at 37 °C under 5% CO_2_. Cells were rinsed twice with ice‐cold PBS (pH 7.4) to terminate CCCP activity. Mitochondrial membrane potential (ΔΨm) was assessed using the JC‐1 MitoMP Detection Kit, purchased from Dojindo (Japan). 1 mL of JC‐1 working solution (5 µg/mL in assay buffer) was applied per chip for 20 min at 37 °C. Unbound dye was removed by three 5‐min washes with pre‐warmed JC‐1 staining buffer. Cells were subsequently maintained in 2 mL of complete culture medium for immediate imaging. Fluorescence signals were captured using a Zeiss LSM 980 confocal microscope (Carl Zeiss AG, Jena, Germany).

### 4.14 RNA Extraction and RT‐qPCR

Following volutrauma and barotrauma treatment, OPECs and PMECs in the chips were separately harvested, washed once with ice‐cold phosphate‐buffered saline (PBS, pH 7.4), and lysed in TRIzol Reagent (Thermo Fisher Scientific, Waltham, MA, USA; Cat# 15596026) for total RNA extraction. RNA purity and concentration were determined spectrophotometrically (NanoDrop 2000, Thermo Fisher Scientific) with A260/A280 ratios between 1.8 and 2.0. Genomic DNA contamination was eliminated using HiScript III RT SuperMix for qPCR (+gDNA wiper, Vazyme Biotech, Nanjing, China; Cat# R323‐01) during reverse transcription of 1 µg RNA into cDNA. Reverse Transcription Quantitative Polymerase Chain Reaction (RT‐qPCR) was performed using a CFX96 Touch Real‐Time PCR Detection System (Bio‐Rad Laboratories, Hercules, CA, USA) with ChamQ Universal SYBR Green Master Mix (Vazyme Biotech; Cat# Q711‐02). Reactions (10 µL total volume) contained 10 ng cDNA and 200 nM gene‐specific primers (Table S2). Thermocycling conditions: initial denaturation at 95 °C for 30 sec; 40 cycles of 95 °C for 10 sec and 60 °C for 30 sec. Melt curve analysis (65‐95 °C, increment 0.5 °C/5 sec) confirmed primer specificity. Relative gene expression was calculated via the 2−ΔΔCt method, normalized to the endogenous reference gene β‐actin (primer efficiency validated at 90–110%).

### 4.15 RNA‐Seq Analysis

Total RNA was isolated from volutrauma‐ and barotrauma‐treated pulmonary acinar epithelial cells and microvascular endothelial cells using the RNeasy Mini Kit (Qiagen, Hilden, Germany). Polyadenylated mRNA was subsequently enriched via the Poly(A) mRNA Magnetic Isolation Module (New England Biolabs, Ipswich, MA, USA). RNA integrity was verified using an Agilent 2100 Bioanalyzer (Agilent Technologies, Santa Clara, CA, USA; RIN > 8.0), while mRNA concentration was quantified with the Qubit RNA High Sensitivity Assay Kit (Thermo Fisher Scientific, Waltham, MA, USA). Sequencing libraries were prepared from 20 ng of high‐quality mRNA using the NEBNext Ultra II RNA Library Prep Kit (New England Biolabs), followed by fragmentation and adapter ligation. Paired‐end sequencing (2 × 150 bp) was performed on an Illumina NovaSeq 6000 platform (CapitalBio Technology, Beijing, China), generating > 40 million reads per sample. Raw sequencing data were processed through the miARma v1.7.3 pipeline [52]. Adapter‐contaminated and low‐quality reads (Phred score < 30) were trimmed using Cutadapt v2.1 [53], and FastQC v0.11.5 [54] was employed for quality control. Clean reads were aligned to the human reference genome (hg19, UCSC) using STAR v2.5.3a [55] with default parameters. Transcript abundance was quantified as reads per kilobase per million mapped reads (RPKM) via the edgeR v3.24.3 package [56], which also identified differentially expressed genes (DEGs; thresholds: |log2 fold change| > 1, false discovery rate < 0.05).

### 4.16 scRNA‐Seq Analysis

Single‐cell suspensions from volutrauma‐ and barotrauma‐treated pulmonary acinar epithelial cells and microvascular endothelial cells were processed using the Chromium Single Cell 3′ v3.1 Library Preparation Kit (10x Genomics, Pleasanton, CA, USA) following the manufacturer's protocol. Captured cells were lysed, and full‐length complementary DNA (cDNA) was synthesized with cell‐specific barcodes and unique molecular identifiers (UMIs). cDNA amplification was performed via 12‐cycle PCR, and sequencing libraries were constructed by enzymatic fragmentation, end‐repair, adapter ligation, and 10‐cycle sample index PCR. Libraries were sequenced on the Illumina NextSeq 500 (Illumina, San Diego, CA, USA) using 28 cycles (Read 1), 55 cycles (Read 2), and 8 cycles for index reads, or on the Illumina NovaSeq 6000 S1 (Illumina) using 28 cycles (Read 1), 64 cycles (Read 2), and 8 index cycles. Sequencing achieved a median depth of 36,000 reads per cell, with a median sequencing saturation of 65% and an average of 5,000 genes detected per cell. Library quality was validated using an Agilent 2100 Bioanalyzer (Agilent Technologies, Santa Clara, CA, USA), with all samples demonstrating a distinct cDNA size distribution (300‐6,000 bp) and minimal adapter dimer contamination (< 5%).

### 4.17 Statistical Analysis

Raw experimental data were collated using Microsoft Excel (v16.0, Microsoft Corp., Redmond, WA, USA). Statistical analysis was conducted in GraphPad Prism (v8.0, GraphPad Software, San Diego, CA, USA). Intergroup differences between two independent cohorts were assessed via unpaired two‐tailed Student t‐tests, assuming unequal variances. For multi‐group comparisons, one‐way analysis of variance (ANOVA) was applied, followed by Bonferroni's post hoc correction to control family‐wise error rates. All data shown in graphs represent mean ± S.D. Data represent three independent biological experiments unless otherwise stated; ^*^ *p* < 0.05, ^**^ *p* < 0.01, ^***^ *p* < 0.001.

## Author Contributions

Conceptualization: Q.L. and H.L. Methodology: H.L., L.L., Z.H., S.F., R.R. and Z.L. Investigation: S.L., M.Z., W.S. and Z.Z., Visualization: H.L. Supervision: Q.L. Writing – original draft: Q.L., L.L. and H.L. Writing – review & editing: Q.L., L.L. and H.L.

## Funding

Prevention and Control of Emerging and Major Infectious Diseases‐National Science and Technology Major Project 2026ZD01999600/2026ZD01999617, Major Project of Guangzhou National Laboratory (GZNL2023A03004), National Key R&D Program of China (2024YFF0509000) and the National Natural Science Foundation of China (52473108).

## Conflicts of Interest

The authors declare no conflict of interest.

## Supporting information




**Supporting File 1**: advs77244‐sup‐0001‐SuppMat.docx.

**Supporting File 2**: advs77244‐sup‐0002‐Video1.mp4.

**Supporting File 3**: advs77244‐sup‐0003‐Video2.mp4.

**Supporting File 4**: advs77244‐sup‐0004‐Video3.mp4.

## Data Availability

The data that support the findings of this study are openly available in the National Genomics Data Center at https://ngdc.cncb.ac.cn/gsa/, reference number HRA012551.
