# Supplementary material for: A Biofabricated Human Acinus‐on‐a‐Chip Unveils Mechanotransductive Drivers of Ventilator‐Induced Lung Injury via Decoupling Volutrauma and Barotrauma
Source: Adv Sci (Weinh). 2026 Aug 17;13(62):e77244. doi: 10.1002/advs.77244 (PMC13479596; doi:10.1002/advs.77244)
Supplement: Supplementary file 1 [file ADVS-13-e77244-s002.docx]

Supporting Information

A Biofabricated Human Acinus-on-a-Chip Unveils Mechanotransductive Drivers of Ventilator-Induced Lung Injury via Decoupling Volutrauma and Barotrauma

Heng Lu^1,2^, Liansheng Liu^1,2^, Zengyong Hu^1^, Shuxian Fang^2^, Ruohan Ren^2^, Sidi Liu^2^, Meng Zhao^3^, Zhiting Luo^1^, Ziyue Zhang^2^, Wenlong Shao^4^, Qian Liu^1,2*^

**Figure S1. Bioengineered acinus-on-a-chip and integrated pressure control system. a-e**. Hierarchical dimensions of multi-layered acinar-on-a-chip components, including microchannel height (1 mm), epithelial chamber diameter (2 mm), and stromal compartment depth (3 mm), optimized to recapitulate alveolar-capillary interface topology. **f**. Representative images of porous PDMS membranes (10 µm thickness, 7 µm pore diameter, scale bar: 100 µm), enabling polarized epithelial-endothelial crosstalk. **g**. Uniform non-porous PDMS membranes (20 µm thickness, scale bar: 100 µm) for basal mechanical stretch transduction. **h**. Fully bonded multi-layered chip assembly via oxygen plasma treatment and thermal annealing. **i**. Integrated organ-on-a-chip workflow featuring: (i) microfluidic perfusion culture module, (ii) computer-controlled cyclic pressure system (±900 mbar, 0-1 Hz), and (iii) real-time phase-contrast and epifluorescence imaging.

**Figure S2. Quantitative biomechanical profiling and biocompatibility validation of the acinus-on-a-chip model. a-b**. Laser profilometry analysis of 3D hemispherical deformation of acinus-on-a-chip during cyclic pneumatic actuation (-70 mbar, 0.2 Hz), demonstrating physiological morphodynamics of the chip. **c**. Bright-field and CLSM images of epithelial E-cadherin, green)-endothelial (VE-cadherin, red) barrier. Cell nuclei were stained with DAPI (blue). Scale bars: 100 μm, 20 μm. **d**. 3D CLSM of the epithelial-endothelial bilayer barrier. Scale bar: 50 μm. **e**. Transmission electron microscopy (TEM) of lung organoid ultrastructure, highlighting indicated cell subtypes in organoids. Scale bar: 2 µm. **f**. Immunofluorescence staining confirming alveolar type II cell identity via CD63^+^ vesicular localization. Scale bar: 20 µm. **g**. Phase-contrast microscopy of epithelial-endothelial barrier and dimensional changes (R1-R6) of specified ranges under cyclic stretch (-70 mbar, 0.2 Hz), confirming stable monolayer integrity and good circularity. Scale bars: 200 µm.

**Figure S3. Effects evaluation in OPECs and PMECs within the acinus-on-a-chip under volutrauma and barotrauma.** **a**. RT-qPCR analysis of mechanosensitive genes in PMECs: apoptotic effectors (*BAK, ATG9B, FAS*) and cell cycle controllers (*MCM5, MCM6, CDKN1A*), normalized to *GAPDH* with n=3 biological replicates. **b-c**. Quantitative analysis of the indicated protein expression under direct volutrauma and barotrauma exposure in OPECs. **d**. Flow cytometry showing DNA content (PI staining) in OPECs under volutrauma and barotrauma. Data represent mean ± S.D. The data were analyzed by one-way ANOVA with Tukey’s multiple comparisons test (a, b, c). **p* < 0.05, ***p* < 0.01, ****p* < 0.001, ns, not significant.

**Figure S4.** **RNA-seq analysis of PMECs subjected to volutrauma and barotrauma within the acinus-on-a-chip****.** **a**. Hierarchical clustering heatmap of differentially expressed genes (DEGs) in PMECs under volutrauma (V_-70_, V_-200_) versus static conditions. **b-c**. Gene Ontology (GO) enrichment of DEGs in V_-200_-treated PMECs: upregulated biological pathways (b) and downregulated biological processes (c). **d**. GSEA of signaling pathways enriched in V_-200_-treated PMECs versus static controls. **e**. Heatmap of DEGs between static and barotrauma-treated PMECs (B_+20_, B_+40_). **f-g**. GO enrichment analysis of DEGs in B_+40_-treated PMECs: upregulated pathways (f) and downregulated processes (g). **h**. GSEA profiles of enriched signaling pathways in B_+40_-exposed PMECs versus static control.

**Figure S5. scRNA-seq** **analysis of the** **OPECs and PMECs subjected to** **volutrauma and** **barotrauma in acinus-on-a-chip. a-b**. UMAP visualization of 38,511 cells were grouped into 2 distinct clusters (EPCAM^+^CDH1^+^ epithelium and PECAM1^+^CDH5^+^ endothelium) based on curated cell type markers. **c**. Heatmap showing marker genes expressions across indicated 11 cell clusters. **d**. UMAP visualization of the indicated lineage populations in V_-200_- and B_+40_-treated OPECs and PMECs, with comparative static control. **e**. Bar graph displaying the varied distribution of cell types across indicated treatment in OPECs. **f**. GO enrichment analysis of the differential genes in cell cluster of Basal/Sec Trans_c in B_+40_ versus static controls. **g**. Heatmap of cell-cell communication showing different strength of interactions for TGF-β signaling in static, V_-200_, and B_+40_. **h**. Immunoblotting analysis of type I collagen (COL1A1), a key fibrotic matrix protein, in OPECs in the absence and presence of TGF-β1, without and with the treatment of B_+0_ in the acinus-on-a-chip, demonstrating enhanced COL1A1 protein expression under barotrauma. HSP90 served as a loading control. **i**. RT-qPCR validation of fibrotic signature genes in OPECs subjected to indicated treatments. Data are presented as mean ± S.D., and analyzed by one-way ANOVA with Tukey’s multiple comparisons test (i). ***p* < 0.01, ****p* < 0.001.

**Figure S6**. Immunofluorescence co‑staining of P63 (red) and alpha-1 type I collagen (COL1A1, green) on OPECs before and after barotrauma (B_+40_) exposure in the chip. Nuclei were stained with DAPI (blue). Scale bars: 20 µm.

**Figure S7.** Barrier permeability evaluation by fluorescein sodium before and after pharmacological intervention. 10 μg/mL fluorescein sodium was loaded into the upper chamber, and the lower chamber medium was collected for fluorescence measurement at 490/520 nm (excitation/emission) after 12 h.

**Figure S8. The acinus-on-a-chip VILI model exhibits strong clinical correlation with VILI patient cohorts.** **a.** Box-and-whisker plots illustrate mRNA expression levels of indicated genes across healthy controls and VILI (AV-exposed, COVID-19 ARDS-AV, and ARDS-AV) clinical samples (datasets: GSE163426, GSE237252). The median value is denoted by the central line, with whiskers spanning the minimum-to-maximum data range. **b.** GSEA profiles highlight differentially enriched signaling pathways in ARDS-AV patients versus healthy individuals, emphasizing pathways dysregulated in VILI.

**Figure S9**. **Characterization of tumor-adjacent normal lung and lung adenocarcinoma tissues.** **a.** Representative H&E staining of tumor-adjacent normal lung and lung adenocarcinoma tissues. Scale bars: 200 μm. **b.** Immunofluorescence co-staining of AT2 cell marker pro-SPC (green) and AT1 cell marker RAGE (red) on the indicated lung sections. Nuclei were stained with DAPI (blue). Scale bars: 20 μm.

**Figure S10.** **The acinar chip setup with the humidifier.** **a.** The acinar chip was connected to a simplified humidifier to maintain appropriate humidity during ventilation simulation. **b.** Cell viability of epithelial cells in the acinar chip without and with humidifier connection after positive pressure application (+40 mbar, 4 h).

**Figure S11**. **ESC‑derived alveolar organoids in acinus-on-a-chip.** **a.** RT-qPCR analysis of signature gene expression for AT1 (*AQP5*), AT2 (*SFTPC*, *ABCA3*), basal (*P63*), and club (S*CGB1A1*) cells in ESC-derived alveolar organoids (from Prof. Rong's lab, Southern Medical University, China) and OPECs. **b.** Immunofluorescence staining of ESC‑derived alveolar organoids for AT1 (AQP5), AT2 (pro‑SPC), tight junctions (E‑cadherin), and nuclei (DAPI). Scale bars: 20 μm. **c.** Immunoblotting of DNA damage markers (P21, P53) and mitochondrial fission regulators (DRP1, MFF, FIS1) in ESC-derived alveolar epithelial cells before and after exposure to volutrauma and barotrauma, respectively. HSP90 served as a loading control.

**Table S1. Human lung organoids differentiation medium**

| **Reagent** | **Final concentration** | **Amount** |
| --- | --- | --- |
| Advanced DMEM/F12 | n/a | Top up to 500 mL |
| GlutaMAX | 1% | 5 mL |
| HEPES | 1% | 5 mL |
| Penicillin-Streptomycin | 1% | 5 mL |
| B27 supplement | 1x | 10 mL |
| N2 supplement | 1x | 5 mL |
| Nicotinamide | 5 mM | 1 mL |
| N-acetylcysteine | 1.25 mM | 1.25 mL |
| Y-27632 | 5 μM | 500 μL |
| SB202190 | 5 μM | 125 μL |
| A83-01 | 0.5 μM | 25 μL |
| Noggin | 100 ng/ml | 500 μL |
| R-spondin | 500 ng/ml | 250 μL |
| FGF10 | 20 ng/ml | 100 μL |
| Total | n/a | 500 mL |

**Table S2.** **Reagent and resource**

| **REAGENT or RESOURCE** | **SOURCE** | **IDENTIFIER** |
| --- | --- | --- |
| **Antibodies** | | |
| Anti-CDH1 | Abcam | Cat# ab76013 |
| Anti-CDH5 | Abcam | Cat# ab211327 |
| Anti-P65 | Cell Signaling Technology | Cat# 8242T |
| Anti-Phospho-P65 | Cell Signaling Technology | Cat# 3033 |
| Anti-Caspase-3 | Proteintech | Cat# 19677-1-AP |
| Anti-Cleaved-Caspase-3 | Cell Signaling Technology | Cat# 9664 |
| Anti-P53 | Proteintech | Cat# 10442-1-AP |
| Anti-P21 | Cell Signaling Technology | Cat# 2947 |
| Anti-SPB | Proteintech | Cat# 1008832-11 |
| Anti-CC10 | Proteintech | Cat# 26909-1-AP |
| Anti-AQP5 | Abcam | Cat# ab315855 |
| Anti-active-β-Catenin | Cell Signaling Technology | Cat# 8814S |
| Anti-β-Catenin | Cell Signaling Technology | Cat# 8480T |
| Anti-FISI | Cell Signaling Technology | Cat# 32525 |
| Anti-DRP1 | Cell Signaling Technology | Cat# 5391 |
| Anti-OPA1 | Proteintech | Cat# 27733-1-AP |
| Anti-MFF | Proteintech | Cat# 17090-1-AP |
| Anti-pro-SPC | Merck millipore | Cat# AB3786 |
| Anti-RAGE | R&D Systems | Cat# AF1145-SP |
| Anti-P63 | Abcam | Cat# AB124762 |
| Anti-COL1A1 | Cell Signaling Technology | Cat# 66948T |
| Anti-HSP90 | Proteintech | Cat# 13171-1-AP |
| Anti-GAPDH | Proteintech | Cat# 60004-1-Ig |
| Anti-rabbit IgG, HRP-linked Antibody | Cell Signaling Technology | Cat# 7074 |
| Anti-mouse IgG, HRP-linked Antibody | Cell Signaling Technology | Cat# 7076 |
| Goat anti-Rabbit IgG (H+L) Highly Cross-Adsorbed Secondary Antibody, Alexa Fluor 647 | Invitrogen | Cat# A21245 |
| Goat anti-Rabbit IgG (H+L) Highly Cross-Adsorbed Secondary Antibody, Alexa Fluor 488 | Invitrogen | Cat# A11034 |
| **Chemicals, peptides, and recombinant protiens** | | |
| Advanced DMEM/F12 | Thermo Fisher | Cat# 12634010 |
| Penicillin-Streptomycin | Thermo Fisher | Cat# 15140122 |
| GlutaMAX | Thermo Fisher | Cat# 35050061 |
| HEPES | Thermo Fisher | Cat# 15630080 |
| UC2288 | Selleck | Cat# E1190 |
| Mdivi-1 | Selleck | Cat# S7162 |
| HLY78 | Selleck | Cat# S7274 |
| TrypLE Express | Thermo Fisher | Cat# 12605010 |
| Fluorescein sodium | Sigma-Aldrich | Cat# F6377 |
| Corning® Matrigel® Basement Membrane Matrix Growth Factor Reduced, PhenolRed Free | CORNING | Cat# 356231 |
| **Critical commercial assays** | | |
| HiPure Total RNA Kit | Magen | Cat# R4011-03 |
| HiScript III RT SuperMix for qPCR Kit | Vazyme | Cat# R323-01 |
| ChamQ Universal SYBR qPCR Master Mix | Vazyme | Cat# Q711-02 |
| CCK-8 | DOJINDO | CK04 |

**Table S3.** **Primers for RT-qPCR of mRNAs**

| **Primer** | **Sequence (Forward)** | **Sequence (Reverse)** |
| --- | --- | --- |
| *KI67* | ACGCCTGGTTACTATCAAAAGG | CAGACCCATTTACTTGTGTTGGA |
| *PCNA* | CCTGCTGGGATATTAGCTCCA | CAGCGGTAGGTGTCGAAGC |
| *BID* | TGGGACACTGTGAACCAGGAGT | GAGGAAGCCAAACACCAGTAGG |
| *XIAP* | ACCGTGCGGTGCTTTAGTT | TGCGTGGCACTATTTTCAAGATA |
| *EIF4E3* | GCAAAGGGTGGCGTATGGAA | CCCGATGGTTGCTAACAGC |
| *CLDN4* | GGGGCAAGTGTACCAACTG | GACACCGGCACTATCACCA |
| *MCM5* | ATGTCGGGATTCGACGATCCT | CCAGGTTGTAATGCCGCTTG |
| *MCM6* | GAGGAACTGATTCGTCCTGAGA | CAAGGCCCGACACAGGTAAG |
| *DUSP5* | GGAAAACCAGTGGTAAATGTCAGC | TGATGTGCAGGTTGGCGAGGAA |
| *ATG9B* | ACCCTGTCAGATGCCATCCTAC | CCAGTAGCTGAAGAGGTTGCAG |
| *CDKN1A* | AGGTGGACCTGGAGACTCTCAG | TCCTCTTGGAGAAGATCAGCCG |
| *FAS* | GGACCCAGAATACCAAGTGCAG | GTTGCTGGTGAGTGTGCATTCC |
| *BAK* | TTACCGCCATCAGCAGGAACAG | TTACCGCCATCAGCAGGAACAG |
| *CDCA7* | GGGTGGCGATGAAGTTTCCA | GGGGATGTCTTCCACGGAAC |
| *PMS2* | TATCGGCTCTGTGTTTGGGCAG | AGCATCGGAACAGCTCAAACCG |
| *DDB2* | CCAGTTTTACGCCTCCTCAATGG | GGCTACTAGCAGACACATCCAG |
| *OPA1* | GTGGTTGGAGATCAGAGTGCTG | GAGGACCTTCACTCAGAGTCAC |
| *PINK1* | GTGGACCATCTGGTTCAACAGG | GCAGCCAAAATCTGCGATCACC |
| *WNT4* | AGGAGGAGACGTGCGAGAAA | CGAGTCCATGACTTCCAGGT |
| *WNT7B* | CACAGAAACTTTCGCAAGTGG | GTACTGGCACTCGTTGATGC |
| *AXIN2* | CAACACCAGGCGGAACGAA | GCCCAATAAGGAGTGTAAGGACT |
| *DKK1* | GGTATTCCAGAAGAACCACCTTG | CTTGGACCAGAAGTGTCTAGCAC |
| *DVL1* | GCATAACCGACTCCACCATGTC | GATGGAGCCAATGTAGATGCCG |
| *COL1A1* | GATTCCCTGGACCTAAAGGTGC | AGCCTCTCCATCTTTGCCAGCA |
| *ACTA2* | CTATGCCTCTGGACGCACAACT | CAGATCCAGACGCATGATGGCA |
| *TGFB* | TACCTGAACCCGTGTTGCTCTC | GTTGCTGAGGTATCGCCAGGAA |
| *FN1* | ACAACACCGAGGTGACTGAGAC | GGACACAACGATGCTTCCTGAG |
| *TGFBR2* | GTCTGTGGATGACCTGGCTAAC | GACATCGGTCTGCTTGAAGGAC |
| *ACTIN* | CATGTACGTTGCTATCCAGGC | CTCCTTAATGTCACGCACGAT |
| *AQP5* | TACGGTGTGGCACCGCTCAATG | TACGGTGTGGCACCGCTCAATG |
| *SFTPC* | GTCCTCATCGTCGTGGTGATTG | AGAAGGTGGCAGTGGTAACCAG |
| *ABCA3* | CTTGACAGTCGCAGAGCACCTT | CTCCGTGAGTTCCACTTGTCCT |
| *P63* | CAGGAAGACAGAGTGTGCTGGT | AATTGGACGGCGGTTCATCCCT |
| *SCGB1A1* | GCTGAAGAAGCTGGTGGACACC | GCGTGGACTCAAAGCATGGCAG |

**Movie S1.**

Schematic animation of the structure of each layer of the acinus-on-a-chip.

**Movie S2.**

Immunofluorescence staining of double-layer cells within the acinus-on-a-chip.

**Movie S3.**

Simulate the three-dimensional respiratory movement of alveoli.
